# Radiological, Molecular, and Pathological Factors Unite: A Model for Predicting Recurrence‐Free Survival in Pathological Stage I Lung Adenocarcinoma

**DOI:** 10.1111/1759-7714.70291

**Published:** 2026-04-23

**Authors:** Hui Zeng, Yufei Huang, Zehao Song, Zhenlong Yuan, Jingyu Ren, Jiaxi Xu, Fangzhou Ren, Wenbin Li, Jianming Ying, Changyuan Guo, Wei Tang, Ming Liang, Yuanjie Zhang, Qin Xu, Qi Xue, Fengwei Tan

**Affiliations:** ^1^ Department of Thoracic Surgery National Cancer Center/National Clinical Research Center for Cancer/Cancer Hospital, Chinese Academy of Medical Sciences and Peking Union Medical College Beijing China; ^2^ Department of Immunology and National Key Laboratory of Medical Molecular Biology Institute of Basic Medical Sciences, Chinese Academy of Medical Sciences (CAMS) and Peking Union Medical College Beijing China; ^3^ Department of Oral and Maxillofacial‐Head and Neck Oncology, Ninth People's Hospital Shanghai Jiao Tong University, School of Medicine Shanghai China; ^4^ Department of Pathology National Cancer Center/National Clinical Research Center for Cancer/Cancer Hospital, Chinese Academy of Medical Sciences and Peking Union Medical College Beijing China; ^5^ Department of Diagnostic Radiology National Cancer Center/National Clinical Research Center for Cancer/Cancer Hospital, Chinese Academy of Medical Sciences and Peking Union Medical College Beijing China; ^6^ Department of Thoracic Surgery The First Hospital of China Medical University Shenyang China; ^7^ Shanghai Key Laboratory of Stomatology & Shanghai Research Institute of Stomatology Shanghai China

**Keywords:** lung adenocarcinoma, online algorithm, pathological stage I, prediction model, recurrence‐free survival

## Abstract

**Background:**

The tumor and node metastasis (TNM) staging and pathological grading systems are currently insufficient for accurately predicting recurrence‐free survival (RFS) in patients with pathological stage (p‐stage) I lung adenocarcinoma (LUAD). Therefore, there is an urgent need for a more economical and applicable clinical prediction model to assess the risk of recurrence and guide clinical postoperative care.

**Methods:**

This retrospective study included 544 patients with p‐stage I LUAD who were randomly allocated to development (272 patients) and validation (272 patients) cohorts. Cox regression and backward model selection were used to develop the prediction model. The predictive performance of the model was then compared with that of the current TNM staging system and two major pathological grading systems. The primary endpoint was RFS.

**Results:**

A total of 79 out of 544 patients with p‐stage I LUAD experienced recurrence after surgery. Four risk factors were incorporated into a weighted risk index—high‐grade patterns ratio, epidermal growth factor receptor mutation status, spread through air spaces status and consolidation tumor ratio—to establish the “CEHS” RFS prediction model. This model demonstrated superior predictive accuracy compared with existing staging and grading systems. High‐risk patients had significantly shorter RFS than low‐risk patients did. An online algorithm based on the CEHS model was also developed.

**Conclusion:**

We established and validated a novel model that integrates radiological, molecular and pathological features to predict RFS in patients with p‐stage I LUAD. This new model exhibited excellent discriminatory power for classifying early‐stage LUAD patients at different risks of recurrence.

## Graphical abstract

We developed CEHS, a novel multiparameter model integrating radiological, molecular, and pathological features, which outperforms TNM staging and existing grading systems in predicting recurrence‐free survival for patients with pathological stage I lung adenocarcinoma.

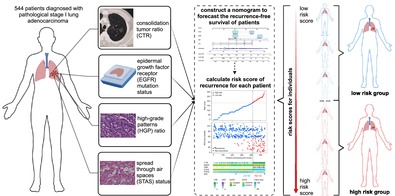

**Abbreviations**
AIC: Akaike's information criterion
AJCC: American Joint Committee on Cancer
AUC: area under the curve
CI: confidence interval
CT: computed tomography
CTR: consolidation tumor ratio
DCA: decision curve analysis
EGFR: epidermal growth factor receptor
FFPE: formalin‐fixation paraffin‐embedding
HGP: high‐grade patterns
HR: hazard ratio
IASLC: International Association for the Study of Lung Cancer grading
IQR: interquartile range
K‐M: Kaplan–Meier
LUAD: lung adenocarcinoma
PPPBG: previous predominant pattern‐based grading
p‐stage: pathological stage
pT: pathological Tumor
pTNM: pathological Tumor‐Node‐Metastasis
RFS: recurrence‐free survival
STAS: spread through air spaces
TNM: tumor and node metastasis

## 1 Introduction

Lung adenocarcinoma (LUAD), the predominant histological subtype of lung cancer, has high global morbidity and mortality [1, 2]. Early detection and treatment significantly improve prognosis [2, 3]. Surgical resection is the standard for stage I LUAD, with a 5‐year survival rate around 80%. However, 10%–30% of patients experience recurrence [4, 5, 6, 7, 8], with 27%–57% succumbing to mortality within 3 years thereafter [9, 10, 11]. Current recurrence prediction remains inadequate. Existing classification systems, including tumor and node metastasis (TNM) stage, the previous predominant pattern‐based grading (PPPBG) and the International Association for the Study of Lung Cancer (IASLC) grading systems, are limited to clinical/pathological diagnosis and lack predictive capacity. A more comprehensive staging system that can integrate multiple prognostic factors is needed for adequate stratification of patients at different risks for recurrence.

Various prognostic factors have been proposed, including the consolidation tumor ratio (CTR) [7, 12, 13, 14, 15], epidermal growth factor receptor (*EGFR*) mutations [6, 16, 17, 18, 19], histological subtypes [20, 21, 22, 23, 24, 25, 26], and spread through air spaces (STAS) status [22, 25, 27, 28, 29, 30, 31]. However, some studies question the independent prognostic value of these parameters [32]. *EGFR* mutation, though common in LUAD (especially in Asia), have inconsistent prognostic implications [6, 16, 17, 18, 19]. STAS, a unique tumor invasion pattern distinct from lymphovascular invasion [33], is reported to be marginally associated with decreased RFS [22, 25, 27, 28, 29, 30, 31]. In 2020, the IASLC introduced a novel histologic classification system for LUAD, which involved determining the most predominant pathological growth pattern along with the proportion of the high‐grade patterns (HGP) [23]. Tumors exhibiting a proportion of the HGP greater than 20% were categorized as high grade and were found to be associated with poor prognosis [21, 23]. Nevertheless, the prognostic importance of HGP in LUAD patients has been incompletely explored [20, 22, 24, 26, 34], and the universal applicability of the 20% cutoff is uncertain. Given the limited predictive power of single factors, relying on them alone is insufficient. While some multivariable models exist, they often focus on advanced LUAD [2, 5, 6, 17, 31, 35], with early‐stage models limited by small sample sizes and short follow‐up. Accurate recurrence risk stratification remains challenging.

To address this problem, we analyzed a retrospective cohort of 544 pathological stage I LUAD patients with complete clinical and follow‐up data. To our knowledge, this retrospective cohort represents one of the largest population‐based studies of p‐stage I LUAD. We developed and validated a multivariable model integrating clinicopathological parameters and molecular parameters, including the CTR, *EGFR* mutation status, HGP ratio, and STAS status, to predict RFS. This model outperformed TNM, PPPBG, and IASLC grading in identifying high‐risk patients.

## 2 Methods

### 2.1 Patient Selection and Information Extraction

A total of 544 p‐stage I LUAD patients who underwent surgical resection at the Department of Thoracic Surgery of the Cancer Hospital, Chinese Academy of Medical Sciences, from January 2014 to June 2018 were included. The inclusion criteria were: (1) pathologically confirmed primary LUAD; (2) postoperative diagnosis of no lymph node metastasis or other site metastasis; and (3) no neoadjuvant therapy. We excluded patients who had (1) adenocarcinoma in situ or minimally invasive adenocarcinoma, (2) incomplete clinicopathological or follow‐up data, or (3) were missing formalin‐fixation paraffin‐embedding (FFPE) tissue blocks.

Baseline information and recurrence status were obtained from medical records and follow‐up (in‐person or by‐phone). Recurrence assessment included physical examination, blood tests, and chest computed tomography (CT). Additional imaging (abdominal CT, brain MRI, bone or PET scans) was performed when recurrence was suspected. Based on these tests, tumor recurrence was diagnosed. RFS was calculated from the date of surgery to recurrence or last contact. Follow‐up was completed on December 24, 2022. Collected variables included age at surgery, sex, smoking history, surgical extent, tumor location, EGFR mutation status, and recurrence outcome.

### 2.2 Study Design

**FIGURE 1:**
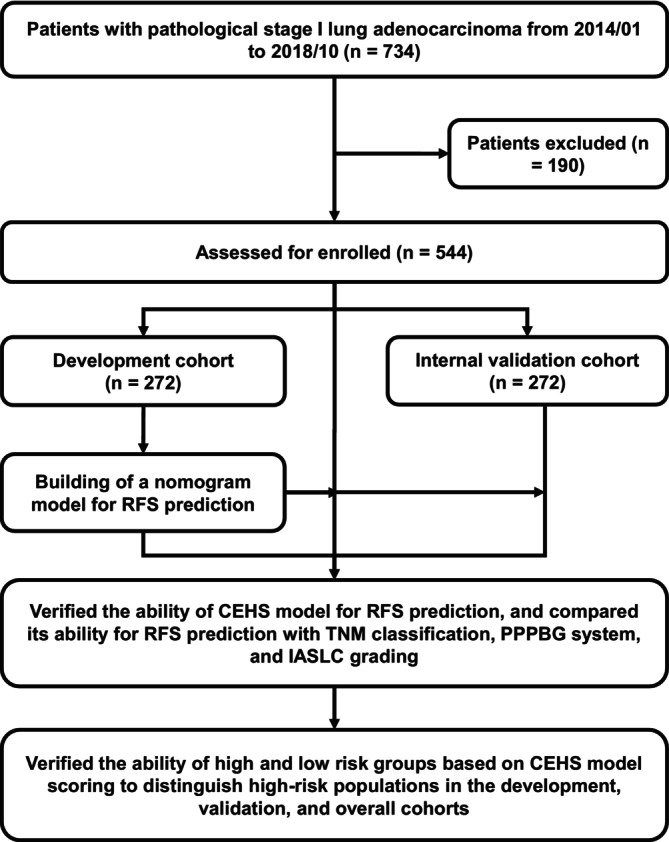
Flowchart of the study design. IASLC, International Association for the Study of Lung Cancer; PPPBG, previous predominant pattern‐based grade; RFS, recurrence‐free survival; TNM, tumor‐node‐metastasis.

As shown in Figure 1, patients were randomly assigned (1:1) to development (*n* = 272) and validation (*n* = 272) cohorts. Multivariable Cox regression was used in the development cohort to identify RFS‐associated risk factors and construct a scoring model. Model performance was then compared with pathological TNM stage (pTNM stage), the PPPBG system, and IASLC grading system across the development, validation, and combined cohorts.

### 2.3 Radiological Features

Preoperative chest CT scans (within 1 week before surgery) were obtained using 64‐detector row scanners (LightSpeed VCT, Discovery CT750 HD, Optima CT660, GE Healthcare, Milwaukee, WI, USA; and Toshiba Aquilion, Toshiba Medical Systems, Japan). The tumor area and invasive tumor size in the area with or without ground‐glass opacities on CT were measured. Two imaging physicians independently evaluated the images, recording CTR in the nodules, spiculation, pleural retraction, and vacuoles. CTR was defined as the ratio of consolidation to total nodule volume and categorized as: CTR = 0 (Figure S1A), 0 < CTR < 0.25 (Figure S1B), 0.25 ≤ CTR < 0.50 (Figure S1C), 0.50 ≤ CTR < 0.75 (Figure S1D), 0.75 ≤ CTR < 1.00 (Figure S1E) or CTR = 1.00 (Figure S1F).

### 2.4 Pathological Features

All tumors were confirmed as primary LUAD by two independent pathologists blinded to clinical data. Resected specimens were formalin‐fixed and stained with hematoxylin and eosin for routine analysis. The subtype percentages of each tumor, including lepidic, acinar, papillary, high‐grade acinar (including cribriform or complex glandular pattern), micropapillary, and solid types, were also recorded according to the 2020 IASLC grading system [23]. HGP included high‐grade acinar (Figure S2A–D), micropapillary (Figure S2E) and solid pattern (Figure S2F). Tumors were graded as: grade 1, lepidic predominant with < 20% HGP; grade 2, acinar/papillary predominant with < 20% HGP; and grade 3, any tumor with ≥ 20% HGP [23]. In addition to qualitative grading, individual HGP ratios were quantitatively assessed.

For clarity, the PPPBG system [36] classifies tumors as low grade (lepidic predominant), intermediate grade (acinar or papillary predominant), and high grade (solid or micropapillary predominant). Moreover, STAS and pleural invasion were also assessed (present/absent). STAS was defined as tumor cells within air spaces in the lung parenchyma beyond the edge of the main tumor (Figure S3A–F) [36]. Pathological T stage (T1a‐c, and T2a) and pTNM stage (IA and IB) were based on the 8th edition of the American Joint Committee on Cancer (AJCC) Staging Manual [37].

### 2.5 *EGFR* Mutation

Next generation sequencing was performed on tissue samples from patients. An *EGFR* mutation in exons 18–21 was considered to indicate a positive mutation (Figure S4A–C) [6].

### 2.6 Statistical Analysis

Categorical variables were analyzed using the *χ* ^2^ test or Fisher's exact test; continuous variables using *t*‐tests or Mann–Whitney *U* tests. RFS was calculated using the Kaplan–Meier (K–M) method and the log‐rank test. Hazard ratio (HR) with 95% confidence interval (CI) were calculated using the Cox regression model. To construct the predictive model, a stepwise backward selection based on Akaike's Information Criterion (AIC) was applied, selecting the model with the lowest AIC. Multicollinearity was assessed using variance inflation factors (VIFs), with VIF > 10 indicating collinearity. A risk score was calculated as:

$$\text{Risk score}=\sum_{i}\text{Coefficient of}\;\left(i\right)\times \text{status}\;\left(i\right).$$

where *i* denotes each variable in the model. Calibration charts were used to graphically depict the degree of overestimation or underestimation associated with the use of our new model. Predictive accuracy at 5 years was assessed by receiver operating characteristic (ROC) curve analysis, with area under the curve (AUC) used to quantify performance. Time‐dependent AUCs were calculated to compare the discriminatory power of our model against other prognostic systems (pTNM stage, PPPBG system, and IASLC grading system). AUC values greater than 0.7 suggest a reasonable estimation. Decision curve analysis (DCA) was performed to evaluate clinical utility. Optimal cutoff values for risk stratification were determined using the maximally selected log‐rank statistic.

All tests were two‐sided with a level of significance set at *p* < 0.05, and borderline significance as 0.05 < *p* < 0.10. The statistical analysis was conducted using R software version 4.2.1 (R statistical calculation project, www.project.org).

## 3 Results

### 3.1 Baseline Patient Characteristics

Table 1 summarizes the baseline characteristics of the all‐combined cohort (544 participants; median [IQR] age: 60 [53–65] years), development cohort (272 participants; median [IQR] age: 60 [53–65] years), and internal validation cohort (272 participants; median [IQR] age: 60 [53–65] years). In the all‐combined cohort, 72.2% were nonsmokers (*n* = 393), and 49.6% harbored EGFR mutations (*n* = 270). At the time of analysis, 79 patients (14.5%) had recurred. Median follow‐up duration was 68.7 months (IQR: 60.1–87.3), 68.5 months (IQR: 59.8–88.5), and 69.1 months (IQR: 60.2–86.3) in the all‐combined, development, and internal validation cohorts, respectively. Notably, no patients received postoperative adjuvant therapy (including chemotherapy or EGFR‐targeted tyrosine kinase inhibitors) during the follow‐up period, consistent with the standard of care for p‐stage I disease during the study interval. The demographic and clinicopathological characteristics of the patients in the development and internal validation cohorts were comparable (all *p* > 0.05).

**TABLE 1:** Baseline characteristics of p‐stage I LUAD in the all‐combined, development and internal validation cohorts.

Variable	All‐combined cohort (*n* = 544)	Development cohort (*n* = 272)	Internal validation cohort (*n* = 272)
Age^a^ ^,^ ^b^	60.0 (53.0, 65.0)	60.0 (53.0, 65.0)	60.0 (53.0, 65.0)
Sex^c^			
Female	315 (57.9)	155 (57.0)	160 (58.8)
Male	229 (42.1)	117 (43.0)	112 (41.2)
Smoking history^c^			
Never	393 (72.2)	202 (74.3)	191 (70.2)
Ever	151 (27.8)	70 (25.7)	81 (29.8)
Scope of operation^c^			
Lobectomy	432 (79.4)	219 (80.5)	213 (78.3)
Sublobar resection	112 (20.6)	53 (19.5)	59 (21.7)
Tumor site^c^			
Upper left	139 (25.6)	68 (25.0)	71 (26.1)
Lower left	64 (11.8)	34 (12.5)	30 (11.0)
Upper right	205 (37.7)	98 (36.0)	107 (39.3)
Right middle	49 (9.0)	23 (8.5)	26 (9.6)
Lower right	87 (16.0)	49 (18.0)	38 (14.0)
Spiculation status^c^			
Absence	169 (31.1)	75 (27.7)	94 (34.6)
Present	374 (68.9)	196 (72.3)	178 (65.4)
Pleural retraction status^c^			
Absence	252 (46.4)	128 (47.2)	124 (45.6)
Present	291 (53.6)	143 (52.8)	148 (54.4)
Vacuole status^c^			
Absence	418 (77.0)	213 (78.6)	205 (75.4)
Present	125 (23.0)	58 (21.4)	67 (24.6)
CTRᶜ			
0	120 (22.1)	65 (23.9)	55 (20.2)
< 25%	64 (11.8)	25 (9.2)	39 (14.3)
25%–50%	32 (5.9)	14 (5.1)	18 (6.6)
50%–75%	55 (10.1)	26 (9.6)	29 (10.7)
≥ 75%	125 (23.0)	67 (24.6)	58 (21.3)
1	148 (27.2)	75 (27.6)	73 (26.8)
EGFR^c^			
Wild	274 (50.4)	148 (54.4)	126 (46.3)
Mutation	270 (49.6)	124 (45.6)	146 (53.7)
8th pT stage^c^			
T1a	63 (11.6)	33 (12.1)	30 (11.0)
T1b	294 (54.0)	140 (51.5)	154 (56.6)
T1c	147 (27.0)	77 (28.3)	70 (25.7)
T2a	40 (7.4)	22 (8.1)	18 (6.6)
8th pTNM stage^c^			
IA	504 (92.6)	250 (91.9)	254 (93.4)
IB	40 (7.4)	22 (8.1)	18 (6.6)
PPPBG system^c^			
High	68 (12.5)	32 (11.8)	36 (13.2)
Intermediate	429 (78.9)	220 (80.9)	209 (76.8)
Low	47 (8.6)	20 (7.4)	27 (9.9)
IASLC grading^c^			
Well	68 (12.5)	32 (11.8)	36 (13.2)
Mediately	333 (61.2)	163 (59.9)	170 (62.5)
Poorly	143 (26.3)	77 (28.3)	66 (24.3)
HGP^a^	0.0 (0.0, 0.2)	0.0 (0.0, 0.2)	0.0 (0.0, 0.2)
STAS^c^			
Absence	327 (60.1)	165 (60.7)	162 (59.6)
Present	217 (39.9)	107 (39.3)	110 (40.4)
Recurrence^c^			
Absence	465 (85.5)	225 (82.7)	240 (88.2)
Present	79 (14.5)	47 (17.3)	32 (11.8)
Follow time^a^ ^,^ ^d^	68.7 (60.1, 87.3)	68.5 (59.8, 88.5)	69.1 (60.2, 86.3)

Abbreviations: 8th, eighth edition; CTR, consolidation tumor ratio; EGFR, epidermal growth factor receptor; HGP, high grade patterns; IASLC, International Association for the Study of Lung Cancer; IQR, interquartile range; LUAD, lung adenocarcinoma; PPPBG, previous predominant pattern‐based grade; p‐stage, pathological stage; pT, pathological Tumor; pTNM, pathological Tumor‐Node‐Metastasis; STAS, spread through air spaces.

^ᵃ^ Median (IQR).

^ᵇ^ Years old.

^ᶜ^ Number (%).

^ᵈ^ Month.

### 3.2 Predictive Variable Screening and Construction of the CEHS Model

**FIGURE 2:**
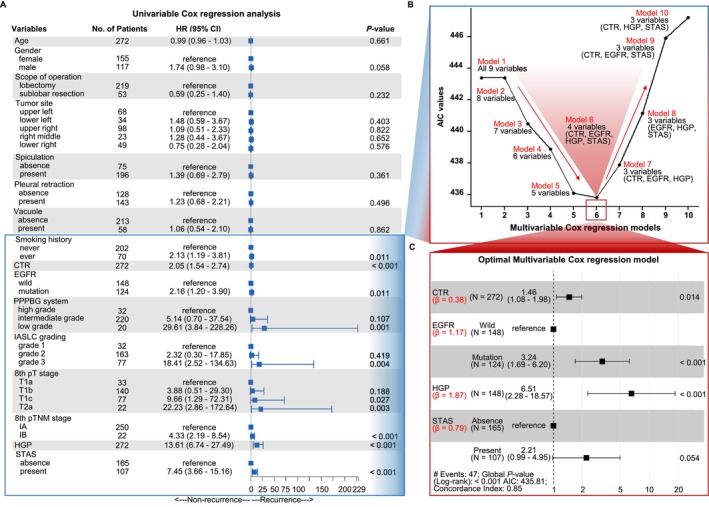
In the development cohort (272 patients), a multivariable Cox regression model was constructed to predict RFS of p‐stage I LUAD, incorporating the variables CTR, EGFR, HGP, and STAS. (A) A forest plot of univariate Cox regression analyses exhibited nine statistically significant risk factors for RFS among 16 clinicopathological variables (*p* < 0.05, respectively). We used these 9 variables (smoking history, CTR, EGFR status, PPPBG system, IASLC grading, 8th pT stage, 8th pTNM stage, HGP ratio, STAS status) to establish models for predicting recurrence of p‐stage I LUAD. (B) A curve chart displaying the AIC values for the different models illustrated the optimal model selection process. The *X*‐axis represents 10 models for predicting p‐stage I LUAD recurrence on the basis of stepdown backward selection: Model 1 (all 9 variables); model 2 (8 variables, excluding variable 8th pTNM stage from Model 1); model 3 (7 variables, excluding variable 8th pT stage from Model 2); model 4 (6 variables, excluding variable IALSC grading from Model 3); model 5 (5 variables, excluding variable PPPBG system from Model 4); model 6 (4 variables, included CTR, EGFR, HGP, and STAS); model 7 (3 variables, including CTR, EGFR, and HGP); model 8 (3 variables, including EGFR, HGP, and STAS); model 9 (3 variables, including CTR, EGFR, and STAS); model 10 (3 variables, including CTR, HGP, and STAS). And model 6 demonstrated the lowest AIC value (435.81) and was deemed the optimal model. (C) A forest plot of the multivariate Cox regression analyses revealed that CTR, EGFR and HGP were significant variables for RFS in the optimal model (*p* < 0.05, respectively). 8th, eighth edition; AIC, Akaike Information Criterion; CTR, consolidation tumor ratio; EGFR, epidermal growth factor receptor; HGP, high grade patterns; IASLC, International Association for the Study of Lung Cancer; LUAD, lung adenocarcinoma; PPPBG, previous predominant pattern‐based grade; p‐stage, pathological stage; pT, pathological Tumor; pTNM, pathological Tumor‐Node‐Metastasis; RFS, recurrence‐free survival; STAS, spread through air spaces.

Through literature review, 16 clinicopathological parameters that could serve as predictors for the recurrence of p‐stage I LUAD were identified (Table 1, Table S1). Given the controversy over the 20% threshold for HGP, both the HGP ratio and IASLC grading (based on this cutoff) were included for analysis. In the development cohort, univariate Cox analysis revealed that smoking history, preoperative CTR, *EGFR* mutation status, the PPPBG system, the IASLC grading system, the 8th pT stage, the 8th pTNM stage, the HGP ratio, and STAS status were significant predictors of recurrence (all *p* < 0.05; Figure 2A).

**FIGURE 3:**
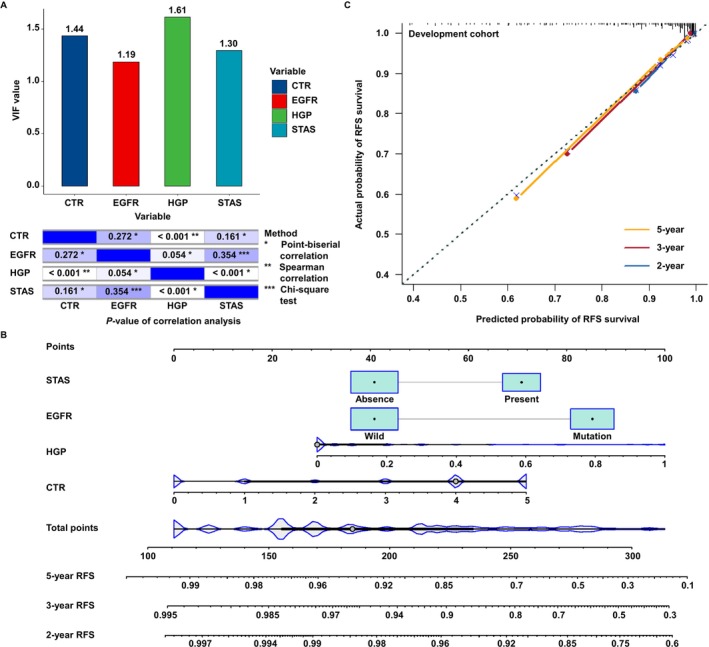
A nomogram was constructed using a multivariable Cox regression model to forecast the RFS of patients with p‐stage I LUAD, and the accuracy of the nomogram was subsequently assessed. (A) The VIF values and correlation *p*‐values of the four variables in the nomogram were examined and found to be non‐collinear (VIF < 2 and/or *p* > 0.05). (B) In the development cohort (272 patients), a novel Cox‐based nomogram was developed and utilized to predict RFS at 1‐, 3‐, and 5‐year by calculating set points based on the scores obtained from the nomogram. (C) A calibration plot demonstrated that our nomogram exhibited satisfactory predictive capability in the development cohort. CTR, consolidation tumor ratio; EGFR, epidermal growth factor receptor; HGP, high grade patterns; LUAD, lung adenocarcinoma; p‐stage, pathological stage; RFS, recurrence‐free survival; STAS, spread through air spaces.

These variables were entered into a multivariable model using backward stepwise selection based on the lowest AIC (Figure 2B; Table S2). Smoking history, pT stage, pTNM stage, IASLC grading, and PPPBG were excluded. The final model included four predictors: the CTR (HR = 1.46, 95% CI = 1.08–1.98), *EGFR* mutation (HR = 3.24, 95% CI = 1.69–6.20), the HGP ratio (HR = 6.51, 95% CI = 2.28–18.57), and STAS (HR = 2.22, 95% CI = 0.99–4.95) (Figure 2C; Table S2). All were independently associated with increased recurrence risk, with the HGP ratio showing the strongest effect (*β* = 1.87), exceeding EGFR mutation (*β* = 1.17), STAS (*β* = 0.79), and CTR (*β* = 0.38). The VIFs for the four variables ranged from 1.19 to 1.61, indicating no multicollinearity (Figure 3A).

Based on the CEHS model coefficients, a nomogram was developed to predict 2‐, 3‐, and 5‐year RFS in p‐stage I LUAD (Figure 3B). Total scores were calculated from individual predictor values. Calibration curves showed strong agreement between predicted and observed RFS in the development cohort (Figure 3C). Furthermore, when the “HGP ratio” parameter was substituted with the “IASLC grading system” in the CEHS model (Figure S5), a notable decrease in the model's predictive accuracy was observed.

### 3.3 Predictive Performance of the CEHS Model

**FIGURE 4:**
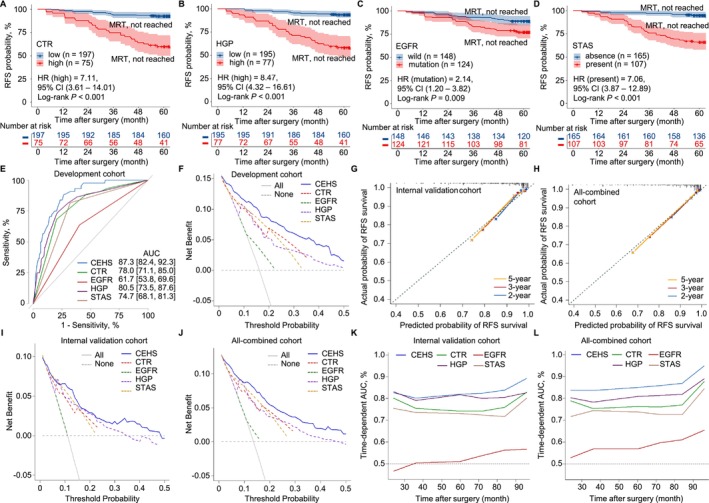
Verified the effectiveness of the CEHS model for patients with p‐stage I LUAD in the development cohort (272 patients), internal validation cohort (272 patients) and all‐combined cohort (544 patients). (A–D) In the development cohort, Kaplan–Meier RFS analysis was employed to generate survival curves and assess the prognostic significance of CTR (*p* < 0.001), HGP (*p* < 0.001), EGFR (*p* = 0.009), and STAS (*p* < 0.001) individually. Patients with high levels of CTR (CTR > 4) or HGP ratio (HGP ratio > 0.15), presence of EGFR mutations, or tumor with STAS had a shorter RFS compared to those with lower levels of CTR (HR_[CTR > 4]_ = 7.11, 95% CI: 3.61–14.01) or HGP ratio (HR_[HGP ratio > 0.15]_ = 8.47, 95% CI: 4.32–16.61), wild‐type EGFR (HR_[EGFR mutation]_ = 2.14, 95% CI: 1.20–3.82), or absence of STAS (HR_[STAS present]_ = 7.06, 95% CI: 3.87–12.89). (E and F) The ROC curve and DCA plot demonstrated that the CEHS model outperformed CTR, EGFR, HGP, and STAS in predicting the risk of recurrence at the 5‐year in the development cohort. (G and H) The calibration plots demonstrated that the performance of our nomogram showed satisfactory predictive power in the internal validation cohort and all‐combined cohort. (I and J) The DCA plots showed the CEHS model was better in predicting the risk of recurrence at 5‐year than CTR, EGFR, HGP, and STAS in the internal validation cohort and all‐combined cohort. And (K and L) time‐dependent AUC values of using the nomogram to predict RFS probability within 8 years in the internal validation cohort and all‐combined cohort. The CEHS model performed best in predicting RFS over time compared with the four prognostic variables. The gray line represents AUC = 0.5, which is considered ideal. AUC, area under the time‐dependent receiver operating characteristics curve; CI, confidence interval; CTR, consolidation tumor ratio; DCA, decision curve analysis; time‐dependent; EGFR, epidermal growth factor receptor; HGP, high grade patterns; HR, hazard ratio; LUAD, lung adenocarcinoma; MRT, median RFS time; p‐stage, pathological stage; RFS, recurrence‐free survival; ROC, receiver operating characteristic; STAS, spread through air spaces; VIF, variance inflation factor.

In the development cohort, RFS was significantly correlated with the CTR, *EGFR* mutation status, HGP ratio and STAS status (Figure 2B and Table S2). Patients with high CTR (CTR > 4) or HGP ratios (HGP ratio > 0.15), *EGFR* mutations, or STAS had shorter RFS than did those with lower CTR (median 60.7 vs. 71.7 months; HR_[CTR > 4]_ = 7.11, 95% CI = 3.61–14.01; *p* < 0.001; Figure 4A); HGP ratios (median 60.3 vs. 76.8 months; HR_[HGP ratio > 0.15]_ = 8.47, 95% CI = 4.32–16.61; *p* < 0.001; Figure 4B); wild‐type EGFR (median 62.5 vs. 82.9 months; HR_[EGFR mutation]_ = 2.14, 95% CI = 1.20–3.82; *p* = 0.009; Figure 4C); or absence of STAS (median 62.5 vs. 72.6 months; HR_[STAS present]_ = 7.06, 95% CI = 3.87–12.89; *p* < 0.001; Figure 4D). The CEHS model demonstrated superior prognostic performance (AUC = 0.873, 95% CI = 0.824–0.923) and outperformed combinations of one to three parameters in both discrimination and net benefit (Figure 4E,F, Figure S6A–D). Calibration in the validation and all‐combined cohorts also showed strong agreement between predicted and observed RFS (Figure 4G,H), with the CEHS model consistently outperforming other combinations and individual predictors (Figure 4I–L, Figure S6E–L). Time‐dependent AUCs confirmed its superior and stable prognostic value (Figure 4K,L).

**FIGURE 5:**
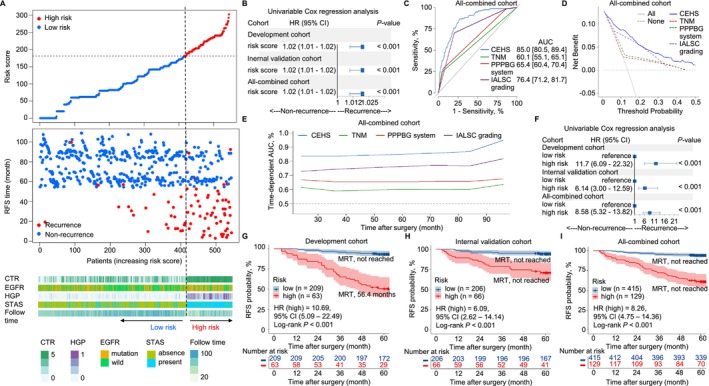
Compared the performance of the CEHS model with the TNM, PPPBG system, and IASLC grading risk classification systems in predicting 5‐year risk of recurrence and patient risk stratification was conducted based on our newly developed risk group. (A) The variables distribution, RFS time and recurrence status of all‐combined cohort (544 patients). The top scatterplot represented the risk‐scores from low to high. Different colors represented different groups (low‐risk group: Risk score ≤ 181; high‐risk group: Risk score > 181). The scatter plot distribution represented the risk‐scores of different samples corresponded to the RFS time and recurrence status. The bottom figure was the variables distribution heatmap. (B) The forest plot of univariate Cox regression analyses was utilized to examine the association of continuous risk scores with risk of recurrence in the development cohort (272 patients), internal validation cohort (272 patients), and all‐combined cohort (544 patients). (C and D) In the all‐combined cohort, the ROC curve and DCA plot showed the CEHS was best at predicting the 5‐year risk of recurrence compared with TNM, PPPBG system, and IASLC grading risk classification systems. (E) Time‐dependent AUC values of using the CEHS model to predict RFS probability within 8 years in the all‐combined cohort. The CEHS performed best in predicting RFS over time compared with the three risk classification systems. The gray line represents AUC = 0.5, which is considered ideal. (F) The forest plot of univariate Cox regression analyses was utilized to compare the risk of recurrence in the high‐risk and low‐risk groups among the three cohorts. (G–I) Kaplan Meier analysis was employed to assess the statistical differences in RFS between the high and low‐risk groups and the results indicated that the high‐risk group exhibited significantly poorer RFS compared to the low‐risk group in all three cohorts (*p* < 0.001). AUC, area under the time‐dependent receiver operating characteristics curve; CI, confidence interval; CTR, consolidation tumor ratio; DCA, decision curve analysis; EGFR, epidermal growth factor receptor; HGP, high grade patterns; HR, hazard ratio; IASLC, International Association for the Study of Lung Cancer; MRT, median RFS time; PPPBG, previous predominant pattern‐based grade; RFS, recurrence‐free survival; ROC, receiver operating characteristic; STAS, spread through air spaces; TNM, Tumor‐Node‐Metastasis.

Recurrence risk scores were calculated by applying the CEHS model to patients in the all‐combined cohort (Figure 5A). Univariate Cox analysis showed a positive correlation between risk score and recurrence risk across all cohorts (HR = 1.02, 95% CI = 1.01–1.02, *p* < 0.001; Figure 5B). Notably, CHES model exhibited superior predictive abilities (AUC = 0.850, 95% CI = 0.805–0.894) compared to three commonly employed tumor classification systems, namely, the 8th pTNM (AUC = 0.601, 95% CI = 0.551–0.651), the PPPBG system (AUC = 0.654, 95% CI = 0.604–0.704), and the IASLC grading system (AUC = 0.764, 95% CI = 0.712–0.817) (Figure 5C). DCA further demonstrated greater clinical utility of the CEHS model (Figure 5D), and time‐dependent AUCs consistently favored CEHS over the three traditional systems (Figure 5E). The prognostic ability of the CEHS compared favorably with those of the 8th pTNM, the PPPBG system, and the IASLC grading system, with AUCs > 0.80 for RFS.

The optimal cutoff point of risk scores for predicting recurrence was determined in patients using maximally selected log‐rank statistics (Figure S7). Subsequently, patients in the development cohort were categorized into high‐risk (risk score > 181) or low‐risk (risk score ≤ 181) populations. An identical cutoff value derived from the development cohort was used to identify individuals at high risk of recurrence within the all‐combined population (Figure 5A). The patient distributions according to our new risk stratification in the development, validation and all‐combined cohorts are reported in Table S3. As expected, the recurrence risk of p‐stage I LUAD was greater in the high‐risk group than in the low‐risk group (development cohort: HR = 11.7, 95% CI = 6.09–22.32; internal validation cohort: HR = 6.14, 95% CI = 3.00–12.59; all‐combined cohort: HR = 8.58, 95% CI = 5.32–13.82; Figure 5F). Correspondingly, survival analysis also suggested notable disparities between the two risk groups in the three cohorts (all *p* < 0.001; Figure 5G–I). The low‐risk population had longer median RFS than the high‐risk group in the development cohort (81.6 months vs. 56.4 months; HR = 10.69, 95% CI = 5.09–22.49; Figure 5G, Table S3), the internal validation cohort (75.3 months vs. 61.6 months; HR = 6.09, 95% CI = 2.62–14.14; Figure 5H, Table S3), and the all‐combined cohort (76.8 months vs. 60.5 months; HR = 8.26, 95% CI = 4.75–14.36; Figure 5I, Table S3).

To conform to our findings, we developed an online algorithm based on our CEHS model that can be used to dynamically predict p‐stage I LUAD RFS (https://zenghui.shinyapps.io/dynnomapp/). In brief, users can easily input each parameter of the CEHS model on the left side of the screen to obtain the RFS probability on the right side (Figure S8).

## 4 Discussion

To our knowledge, the CEHS model, which integrates different types of clinicopathologic and molecular parameters, is the only multiparameter RFS prediction model specific for p‐stage I LUAD patients. A well‐powered cohort, comprehensive screening strategy for clinical and pathological risk factors and long‐term follow‐up enabled use of the CEHS model to accurately stratify the recurrence risk of p‐stage I LUAD patients. Compared to widely employed prognostic tools such as the pTNM stage, the PPPBG system, and the IASLC grading system, this model provides greater net benefit in selecting patients for close postoperative attention at all threshold probabilities.

It is not surprising that CTR and STAS were included as key predictors in the CEHS model. Previous studies have indicated that CTR [7, 12, 13, 14, 15] and STAS status [22, 25, 27, 28, 29, 30] are associated with recurrence risk in LUAD. Ye, Xi and Hattori et al. reported that CTR < 1 was associated with lower recurrence risk in early‐stage LUAD [7, 12, 13, 14, 15]. STAS, recognized by WHO as an additional invasive pattern [23], is an important histologic feature. Caso [25], Dai [27] and Chae [28] et al. demonstrated that STAS provides a plausible explanation for postoperative recurrence in resected LUAD patients. Another three studies [22, 29, 30] revealed that STAS was an independent risk factor for recurrence in patients with resected LUAD. Consistent with these studies, our results support CTR and STAS as independent prognostic factors. However, their individual predictive performances were inferior to that of the CEHS model, indicating limited prognostic utility when used alone.

Notably, the prognostic value of EGFR mutation for RFS in LUAD remains debated. We and others [16, 19] observed poorer RFS in EGFR‐mutant resected LUAD, whereas other studies [6, 17, 18] reported improved RFS, seemingly contradicting our findings. One plausible explanation is the difference in cohort composition between our study and other studies. Our cohort included only p‐stage I patients, whereas Takamochi [6] and D'Angelo [17] included many patients with middle‐advanced disease. EGFR kinase domain mutations, mainly in exons 18–21 [6, 17, 18, 38], lead to constitutive activation of downstream oncogenic pathways (e.g., Shc, STAT3, Akt) [39], promoting tumorigenesis, especially in early‐stage disease. In contrast, advanced‐stage patients often receive chemotherapy or targeted therapy, to which EGFR‐mutant tumors are more responsive [6, 17, 38], potentially improving outcomes. This notion is supported by the results of D'Angelo's experiment, which showed that patients with *EGFR* mutations who did not receive adjuvant tyrosine kinase inhibitors did not have a reduced risk of disease recurrence [17]. Similarly, in multivariate analyses of prognostic factors for RFS, Takamochi's cohort showed that *EGFR* mutation had no statistical significance [6]. Further research is needed to clarify the stage‐specific prognostic role of EGFR mutations. Notably, EGFR mutations are more frequent in lepidic‐pattern tumors [25], which are associated with reduced recurrence in stage I LUAD [21, 23]. Therefore, both EGFR status and histological subtype should be considered when evaluating prognosis. Unlike prior studies [6, 18], our CEHS model integrates these variables, enabling more accurate RFS prediction in p‐stage I LUAD.

The five histological subtypes of LUAD, which were presented in 2011 [20, 36]. entail varying risks of recurrence [20, 21, 23, 24, 26, 34]. LUADs are histologically heterogeneous, with the acinar‐predominant subtype being most common and exhibiting the broadest prognostic range [23]. To improve recurrence risk stratification, a 2020 grading system categorized tumors by high‐grade patterns (HGP), with ≥ 20% HGP defined as poorly differentiated [23]. Jeon et al. [21] supported its utility in predicting recurrence in stage I LUAD. However, the universal applicability of the 20% cutoff remains uncertain. Two studies [26, 34] found that micropapillary/solid‐predominant tumors had worse RFS, while Yanagawa et al. [20] and Choi et al. [24] reported poorer RFS even when these patterns were non‐predominant. Based on these findings, we hypothesized that modeling HGP as a continuous variable could enhance prognostic accuracy and support personalized treatment. Our results confirmed that continuous HGP values outperformed the fixed 20% threshold (IASLC grading) in predicting RFS in p‐stage I LUAD. Excluding IASLC grading in favor of continuous HGP improved model performance. Thus, we propose a novel prognostic approach using continuous HGP values, which warrants further validation. However, we acknowledge that the assessment of an exact continuous HGP ratio may be more susceptible to inter‐observer variability among pathologists than the use of a binary threshold. To ensure the clinical reproducibility of the CEHS model, future implementation may require standardized pathology training or the integration of AI‐assisted pathology to provide objective and precise HGP quantification.

Compared to the widely used pTNM stage [37], PPPBG system [23, 34], and IASLC grading [21, 23, 40] risk assessment tools, the CEHS exhibited greater accuracy and net benefits in predicting the recurrence of p‐stage I LUAD. This may be attributed to its integration of radiological, molecular, and pathological features that are not only directly related to the malignancy of the tumor in the final pathology but also recognized as reliable indicators of tumor mutation burden and invasion [22, 25, 35]. Our findings, consistent with prior studies, underscore the value of combining preoperative and postoperative variables to enhance recurrence risk prediction. Based on the CEHS model, we developed an online prediction tool (https://zenghui.shinyapps.io/dynnomapp/). The aim of this study was to provide an intuitive tool to aid in planning postoperative interventions and management strategies. As all parameters are routinely available, the model is practical for large‐scale implementation without increasing patient burden.

To confirm the accuracy and clinical applicability of our model, we plan to use a multicenter cohort for further verification. Furthermore, considering the economic cost and rapid molecular diagnosis, only *EGFR* was included in this study. In the future, other molecules, such as Ras, will also be evaluated for inclusion in the prediction model.

## 5 Conclusion

We developed and validated a new model, the CEHS, which integrates imaging, *EGFR* mutation status, and pathological parameters to predict RFS in patients with p‐stage I LUAD. Our CEHS model has good clinical utility and has shown greater accuracy in predicting RFS than widely used tools. This knowledge can help clinicians guide the selection of personalized treatment and follow‐up options for p‐stage I LUAD patients.

## Author Contributions

**Jingyu Ren:** data curation. **Jiaxi Xu:** data curation. **Zhenlong Yuan:** data curation. **Yufei Huang:** conceptualization, writing – original draft, writing – review and editing, formal analysis, visualization, investigation, methodology, validation. **Hui Zeng:** conceptualization, writing – original draft, writing – review and editing, data curation, formal analysis, visualization, software, investigation, methodology, validation. **Fangzhou Ren:** data curation. **Zehao Song:** conceptualization, writing – review and editing, visualization, investigation, methodology, writing – original draft, validation. **Jianming Ying:** resources. **Yuanjie Zhang:** data curation. **Wei Tang:** resources. **Ming Liang:** resources. **Wenbin Li:** resources. **Qi Xue:** writing – review and editing, conceptualization, supervision, project administration. **Changyuan Guo:** resources. **Fengwei Tan:** writing – review and editing, funding acquisition, conceptualization, supervision, project administration. **Qin Xu:** formal analysis, writing – review and editing, conceptualization, supervision, project administration.

## Funding

This research was supported by the Capital's Funds for Health Improvement and Research (CFH‐2022‐2‐4025), the National High Level Hospital Clinical Research Funding (2025‐LYZX‐D‐A01), the Noncommunicable Chronic Diseases‐National Science and Technology Major Project (2024ZD0519700/2024ZD0519701), the National Natural Science Foundation of China (82472994, 82541024), the Special Research Central Universities, Peking Union Medical College (2025‐I2M‐C&T‐C‐008), the Fundamental Research Funds for the Central Universities, Peking Union Medical College (3332024216), and the Science and Education Cultivation Fund of the National Cancer and Regional Medical Center of Shanxi Provincial Cancer Hospital (TD2023002).

## Disclosure

The authors have nothing to report.

## Ethics Statement

This is an observational study. The Institutional Review Board of Cancer Hospital, Chinese Academy of Medical Sciences has confirmed that no ethical approval is required.

## Consent

No written consent has been obtained from the patients as there is no patient‐identifiable data included.

## Conflicts of Interest

The authors declare no conflicts of interest.

## Supporting information


**Figure S1:** Typical findings of LUAD were presented on the basis of the findings of computed tomography scan. (A) CTR = 0 was defined as a lung tumor showing only ground glass opacity without solid component. (B‐E) 0 < CTR < 1 was defined as a lung tumor with both ground glass opacity and solid component, whereas (F) CTR = 1 was defined as a tumor showing only consolidation without ground glass opacity. (CTR, consolidation tumor ratio; LUAD, lung adenocarcinoma).

**Figure S2:** Histologic examples of HGP, including (A–D) complex glandular patterns, (E) micropapillary pattern and (F) solid pattern. (HGP, high grade patterns).

**Figure S3:** Histologic example of STAS in lung adenocarcinoma. (STAS, spread through air spaces).

**Figure S4:** Proportion of EGFR 18–21 mutations and wild‐type in (A) the development cohort (*n* = 272), (B) the internal validation cohort (*n* = 272), and (C) the all‐combined cohort (*n* = 544). (EGFR, epidermal growth factor receptor).

**Figure S5:** Comparing the two models, the ROC and DAC results showed that the contribution of HGP continuous variables to the model is greater than that of HGP fixed values (IASLC grading). In (A, B) the development cohort (*n* = 272 patients), (C, D) the internal validation cohort (*n* = 272 patients) and (E, F) the all‐combined cohort (*n* = 544 patients), ROC curves and DAC showed the CEHS model was better in predicting the risk of recurrence at 5‐year than the model (including CTR, EGFR, IASLC grading and STAS). (CTR, consolidation tumor ratio; DCA, decision curve analysis; EGFR, epidermal growth factor receptor; HGP, high grade patterns; ROC, receiver operating characteristic; STAS, spread through air spaces).

**Figure S6:** In the development cohort (*n* = 272 patients), the internal validation cohort (*n* = 272 patients) and the all‐combined cohort (*n* = 544 patients), ROC curves and DAC showed the CEHS model was the best model in predicting the risk of recurrence at 5‐year than (A, B, E, F, I, and J) two‐variables Cox models, and (C, D, G, H, K, and L) three‐variables Cox models. (CTR, consolidation tumor ratio; DCA, decision curve analysis; EGFR, epidermal growth factor receptor; HGP, high grade patterns; ROC, receiver operating characteristic; STAS, spread through air spaces).

**Figure S7:** The optimal cutoff value for risk points based on maximum selection logarithmic rank statistics is 181.

**Figure S8:** An online calculator is available at: https://zenghui.shinyapps.io/dynnomapp/. (CTR, consolidation tumor ratio; EGFR, epidermal growth factor receptor; HGP, high grade patterns; STAS, spread through air spaces).

**Table S1:** The variables included in this study have been reported in previous literature to have prognostic value for patients with LUAD.
**Table S2:** Multivariable Cox regression models for recurrence risk among patients with p‐stage I LUAD in the development cohort based on a stepdown backward selection with Stepwise Removal of Variables (*n* = 272).
**Table S3:** Clinical characteristics in the overall, development, and internal validation cohorts according to our newly developed risk groups.

## Data Availability

The data that support the findings of this study are available from the corresponding author upon reasonable request.
