# Supplementary figures and images for: Radiological, Molecular, and Pathological Factors Unite: A Model for Predicting Recurrence‐Free Survival in Pathological Stage I Lung Adenocarcinoma
Source: Thorac Cancer. 2026 Apr 23;17(8):e70291. doi: 10.1111/1759-7714.70291 (PMC13104728; doi:10.1111/1759-7714.70291)

**A**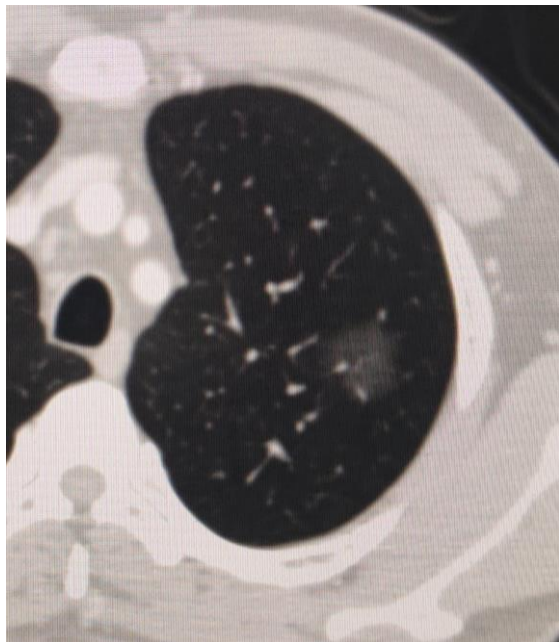

CTR = 0, n = 120

**B**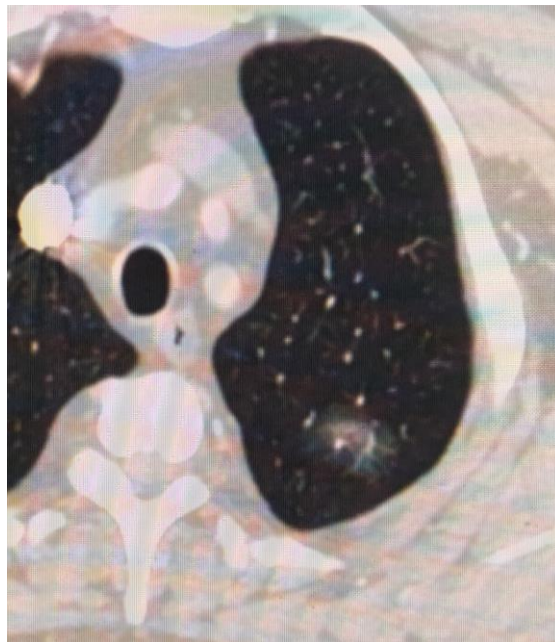

$0 < \text{CTR} < 0.25$ , n = 64

**C**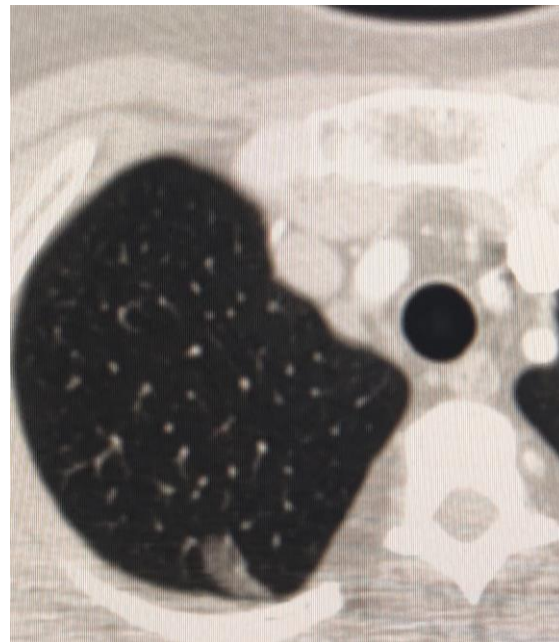

$0.25 \leq \text{CTR} < 0.50$ , n = 32

**D**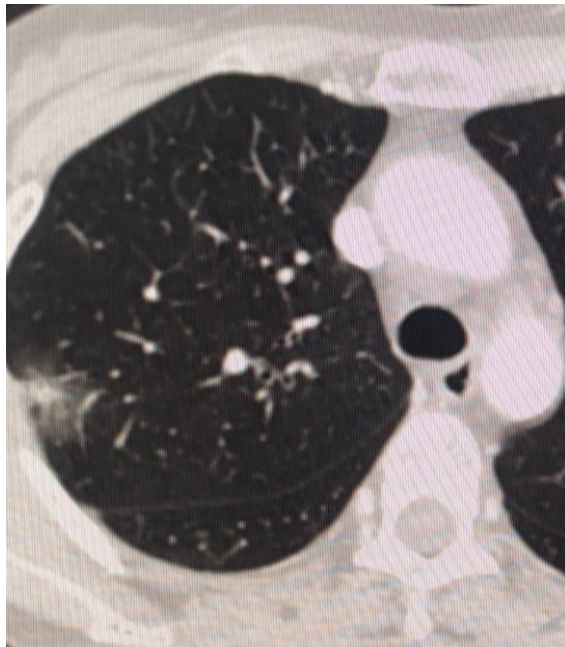

$0.50 \leq \text{CTR} < 0.75$ , n = 55

**E**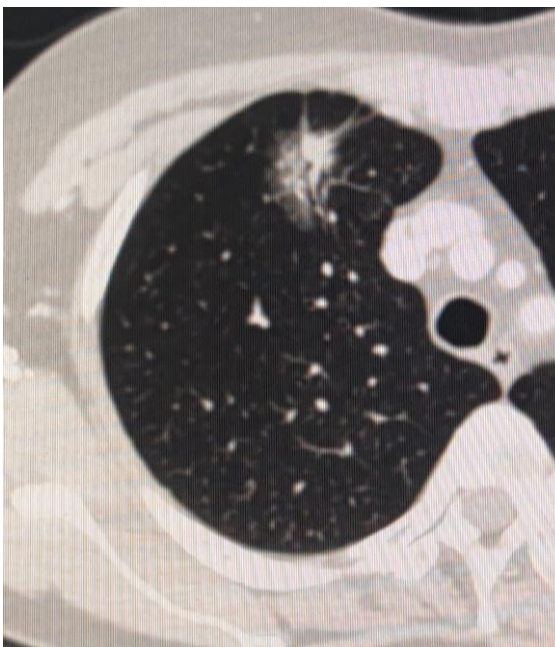

$0.75 < \text{CTR} < 1.00$ , n = 125

**F**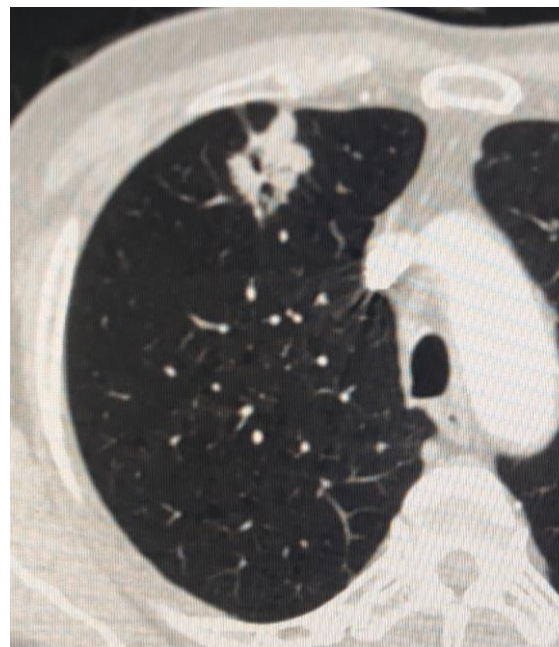

CTR = 1.00, n = 148

Supplement: Supplementary file 1 — Figure S1: Typical findings of LUAD were presented on the basis of the findings of computed tomography scan. (A) CTR = 0 was defined as a lung tumor showing only ground glass opacity without solid component. (B‐E) 0 < CTR < 1 was defined as a lung tumor with both ground glass opacity and solid component, whereas (F) CTR = 1 was defined as a tumor showing only consolidation without ground glass opacity. (CTR, consolidation tumor ratio; LUAD, lung adenocarcinoma). [file TCA-17-e70291-s002.pdf]

**A**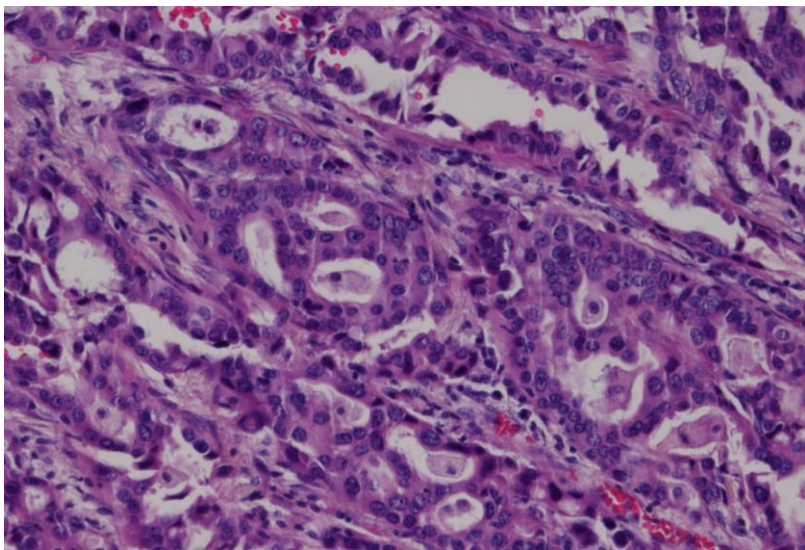**B**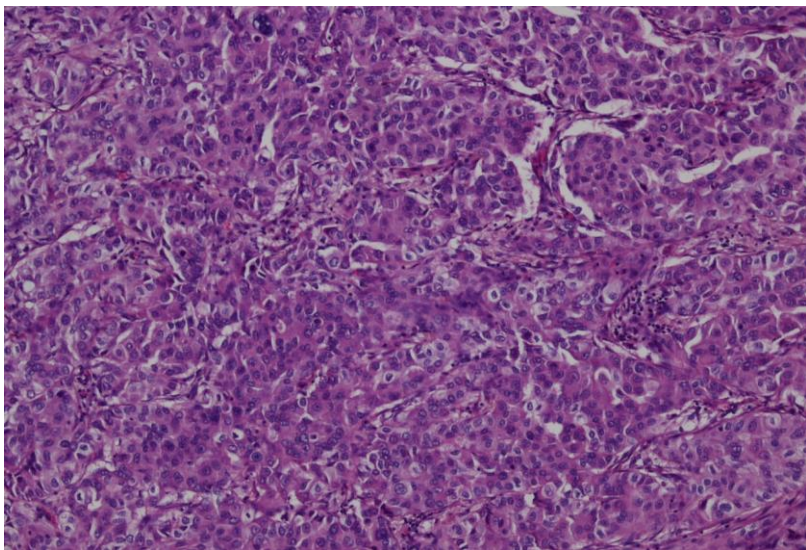**C**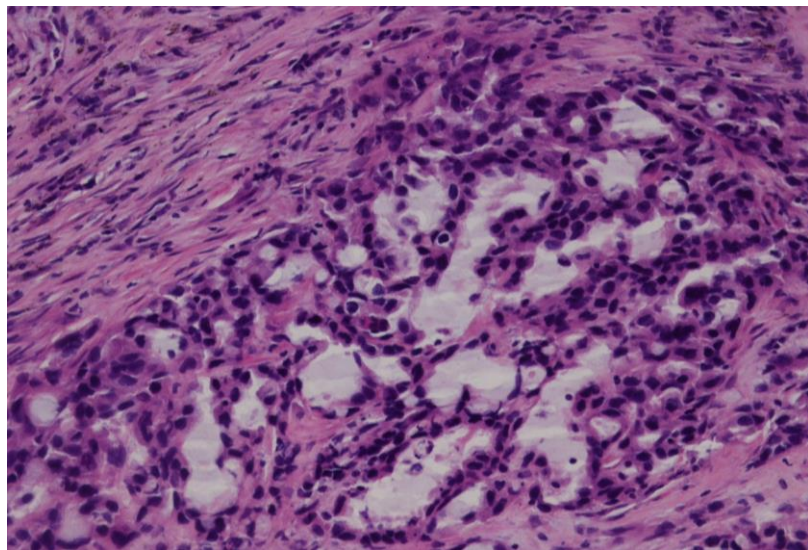**D**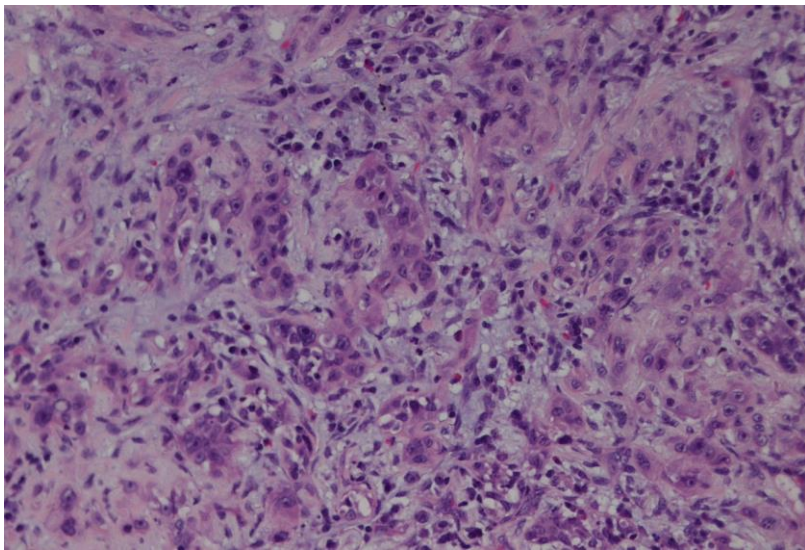**E**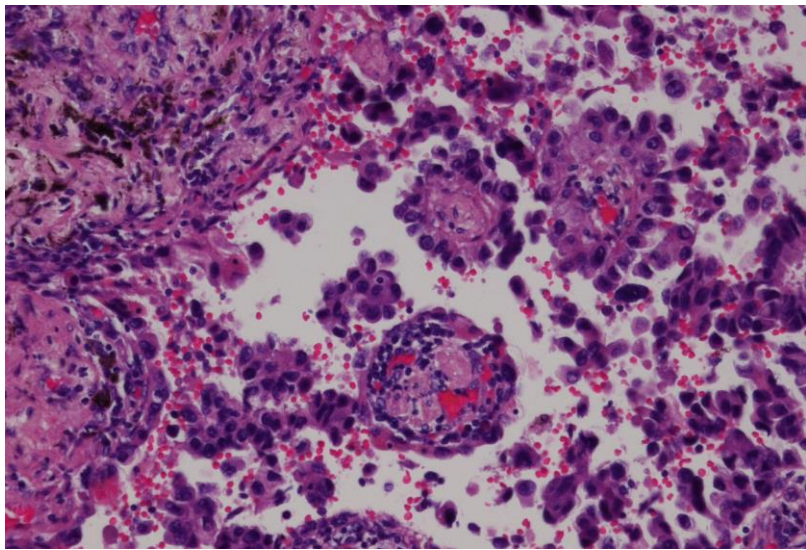**F**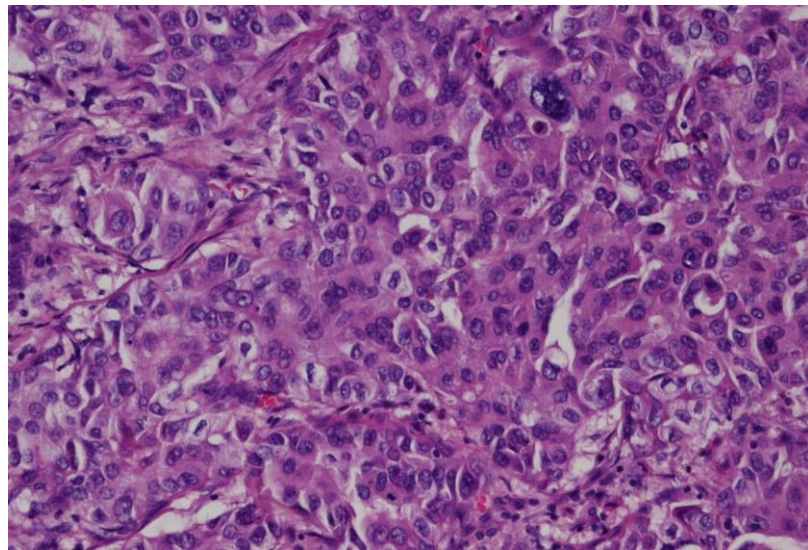

Supplement: Supplementary file 2 — Figure S2: Histologic examples of HGP, including (A–D) complex glandular patterns, (E) micropapillary pattern and (F) solid pattern. (HGP, high grade patterns). [file TCA-17-e70291-s008.pdf]

**A**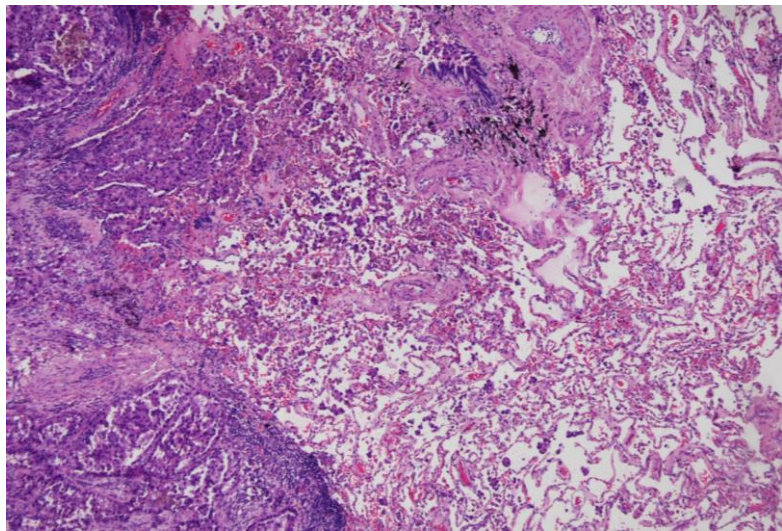**B**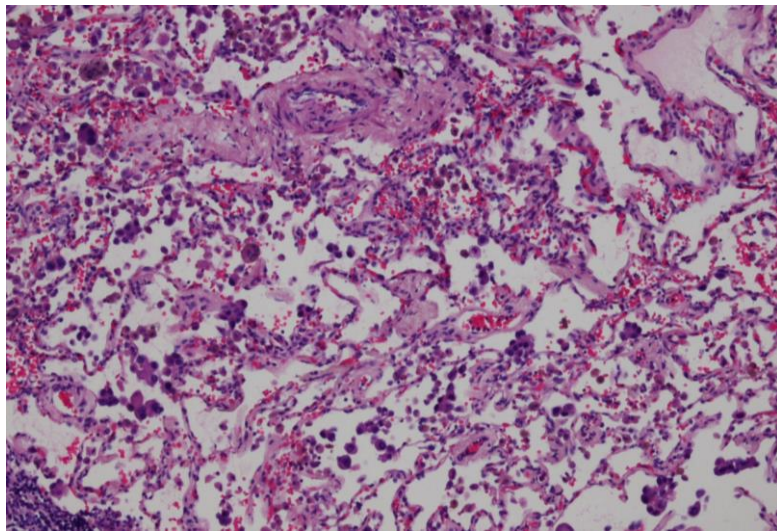**C**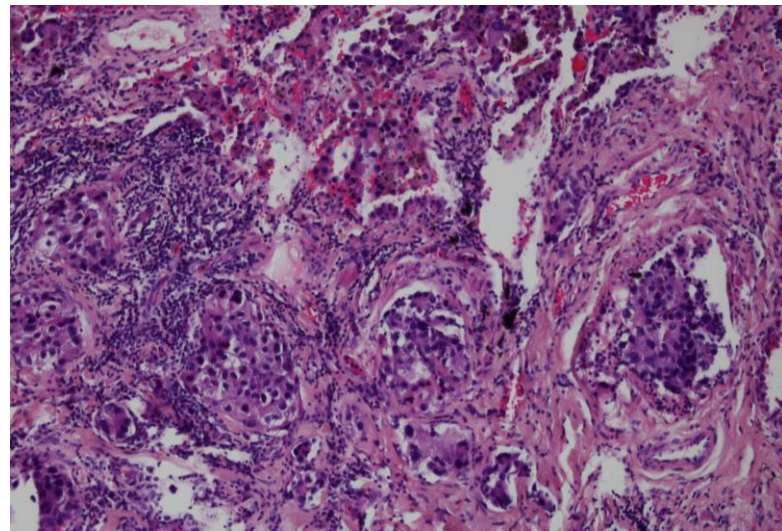**D**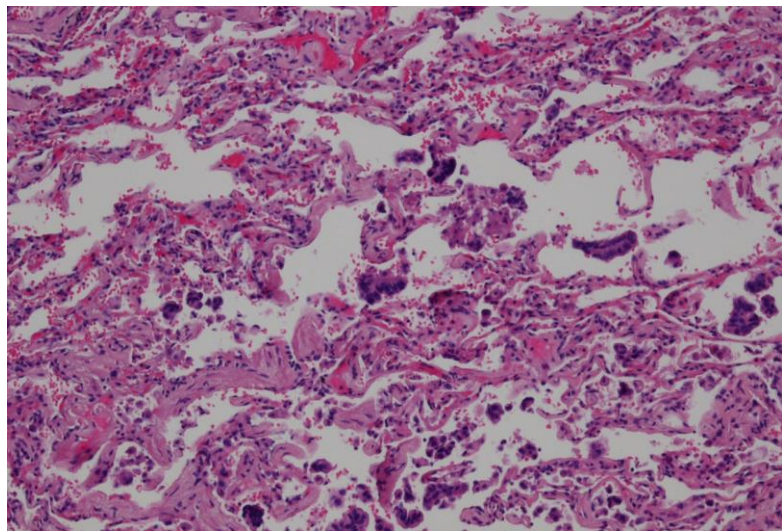**E**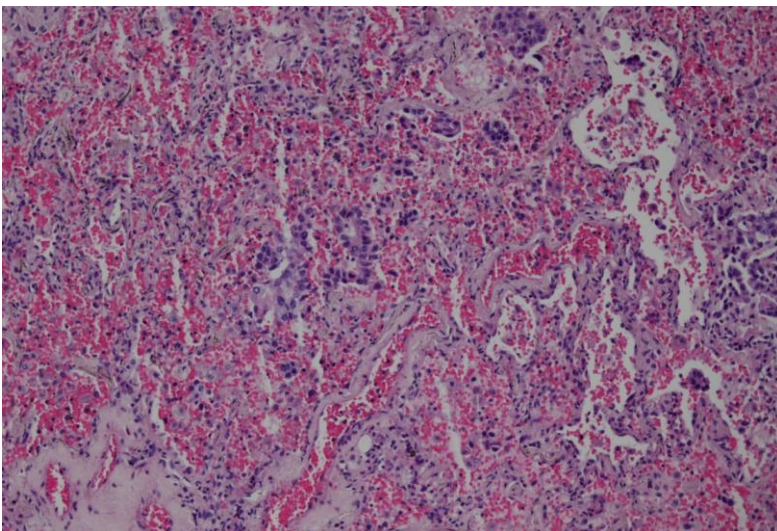**F**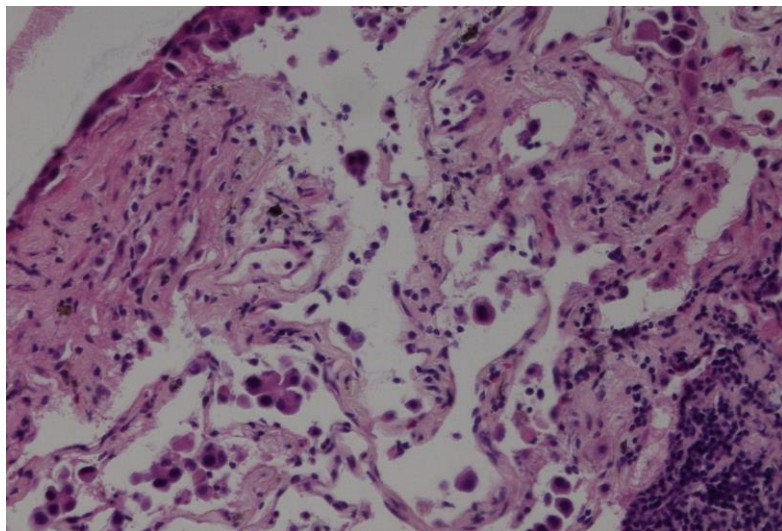

Supplement: Supplementary file 3 — Figure S3: Histologic example of STAS in lung adenocarcinoma. (STAS, spread through air spaces). [file TCA-17-e70291-s009.pdf]

**A****Development cohort**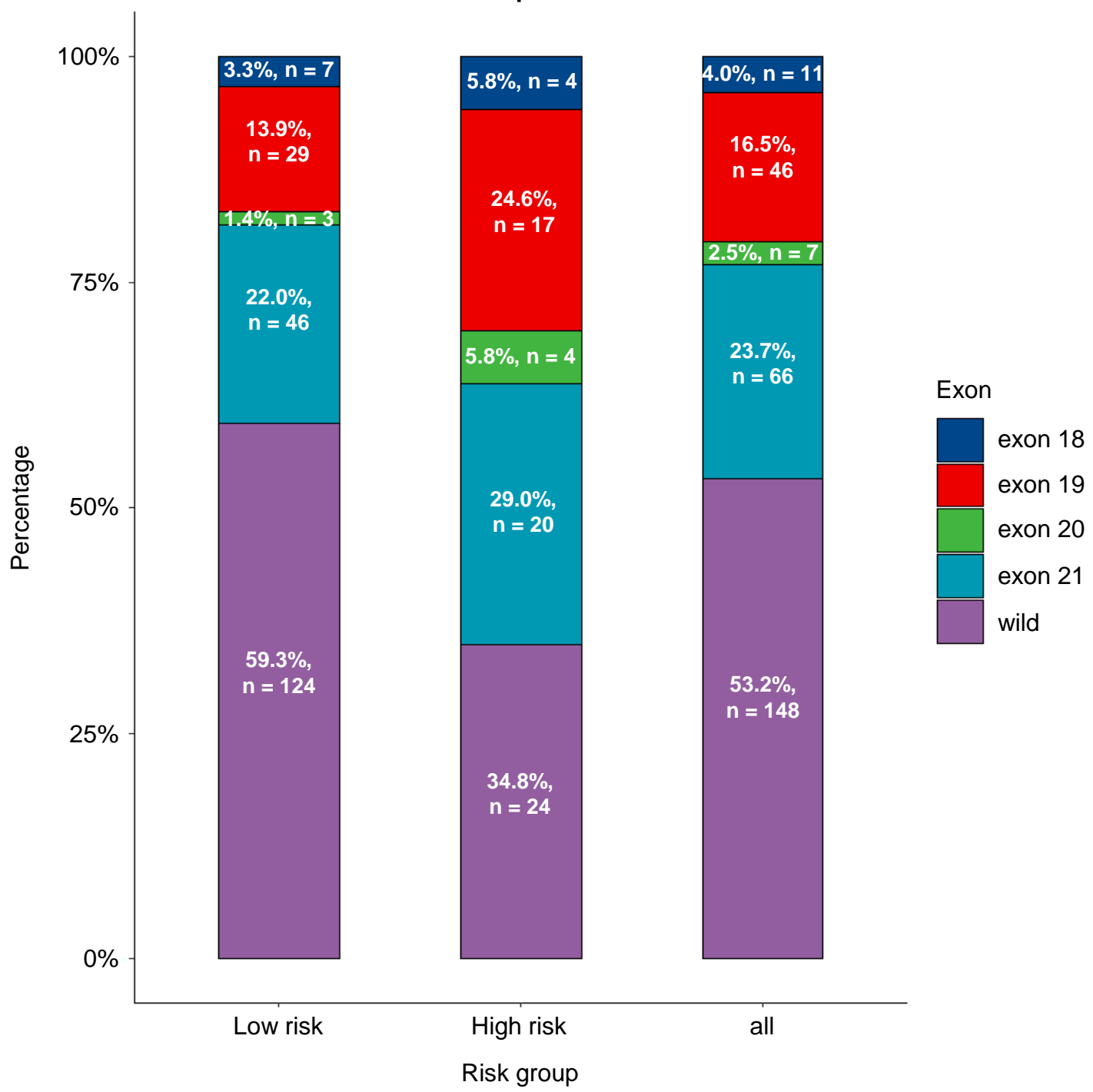**B****Internal validation cohort**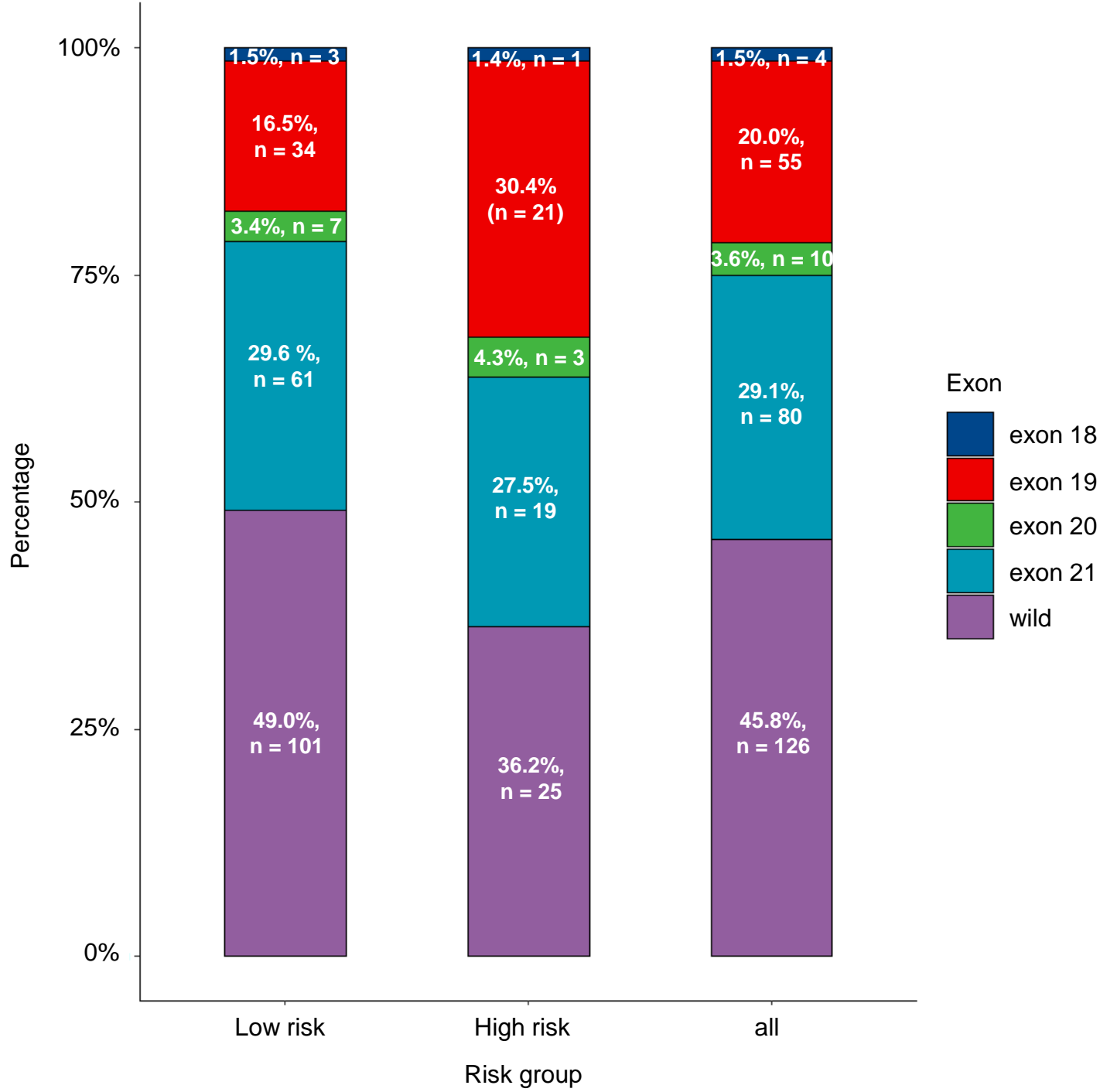**C****All-combined cohort**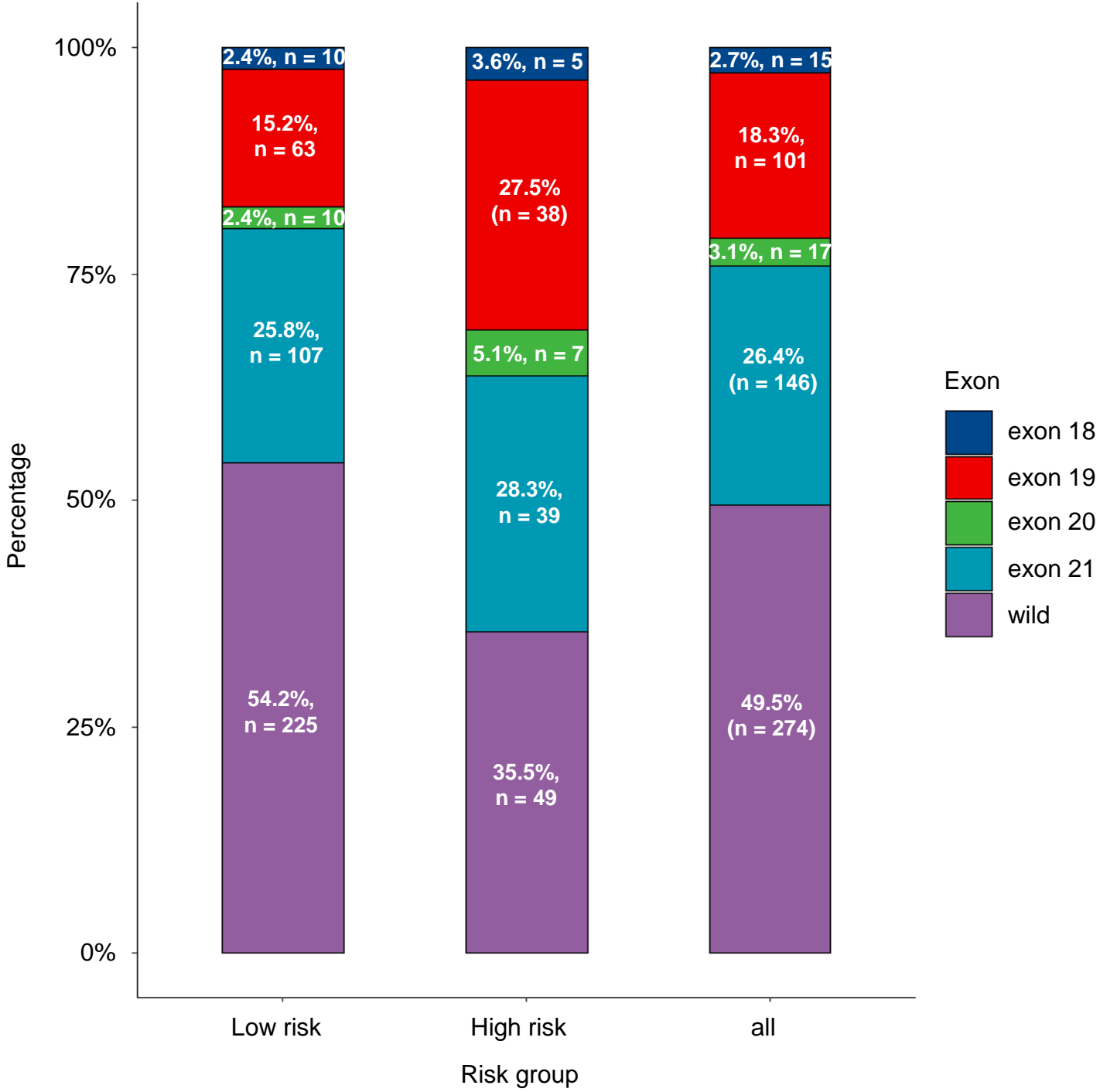

Supplement: Supplementary file 4 — Figure S4: Proportion of EGFR 18–21 mutations and wild‐type in (A) the development cohort (n = 272), (B) the internal validation cohort (n = 272), and (C) the all‐combined cohort (n = 544). (EGFR, epidermal growth factor receptor). [file TCA-17-e70291-s001.pdf]

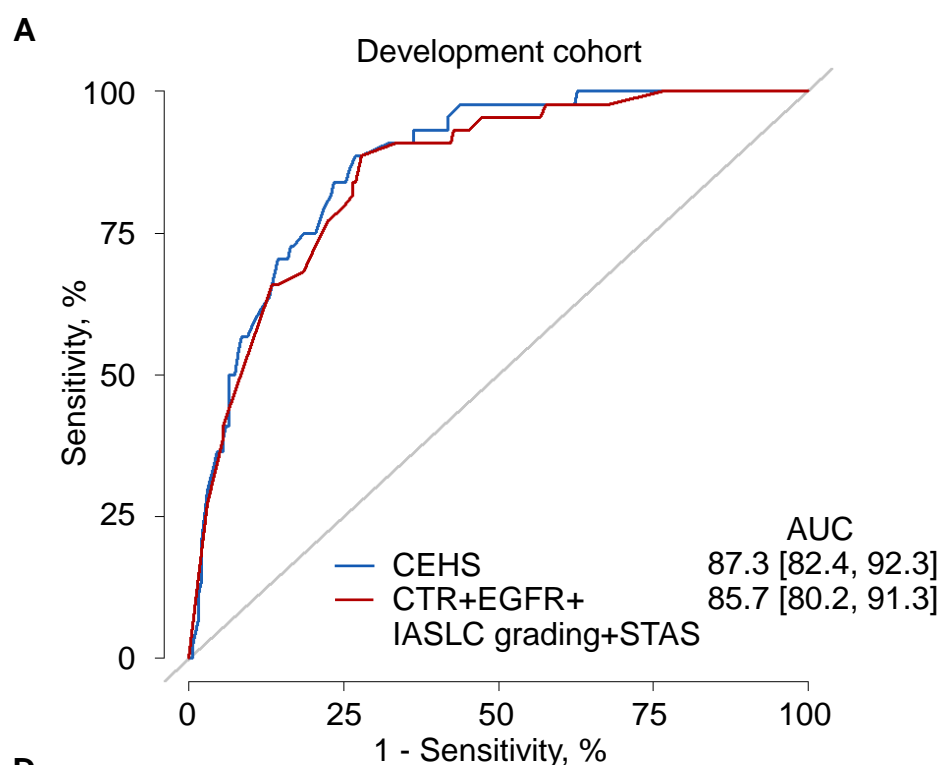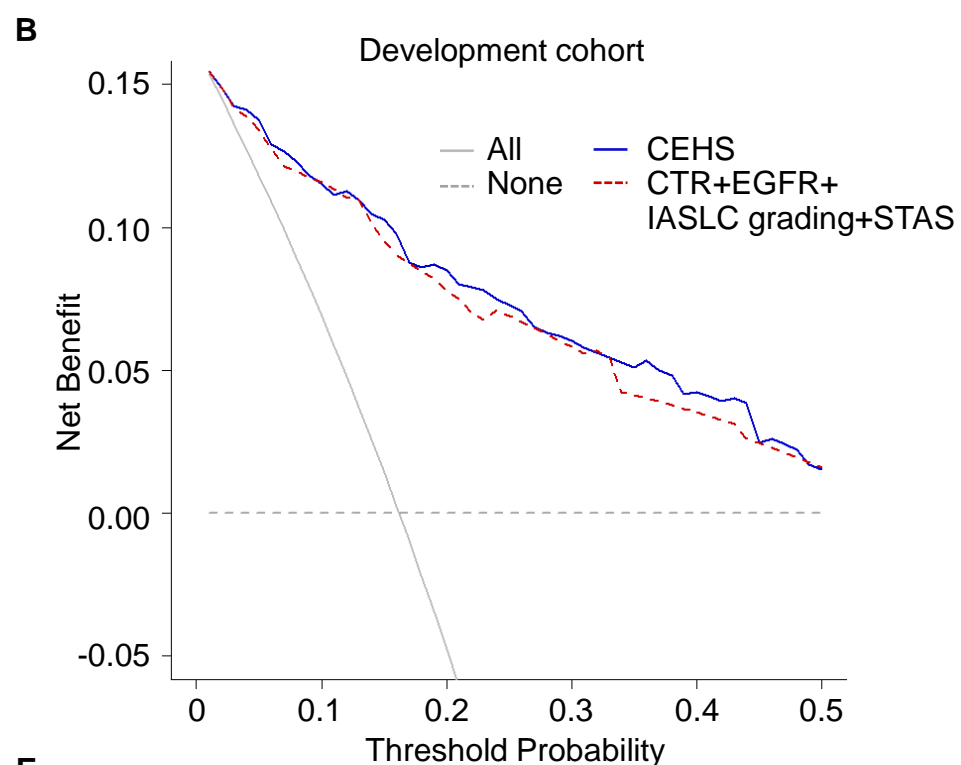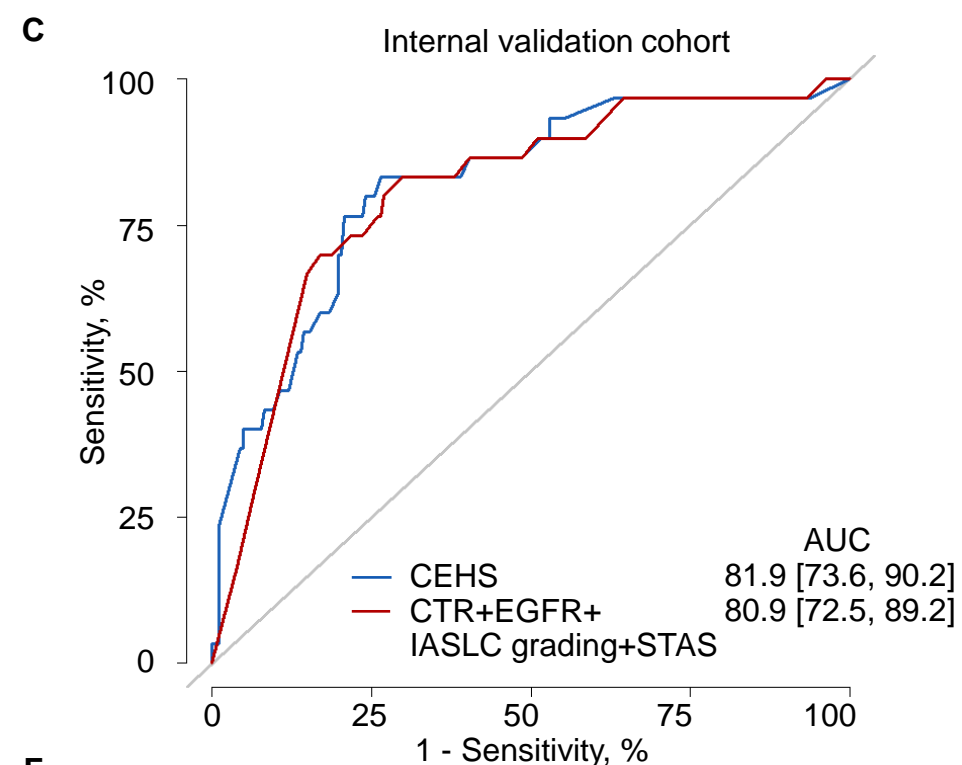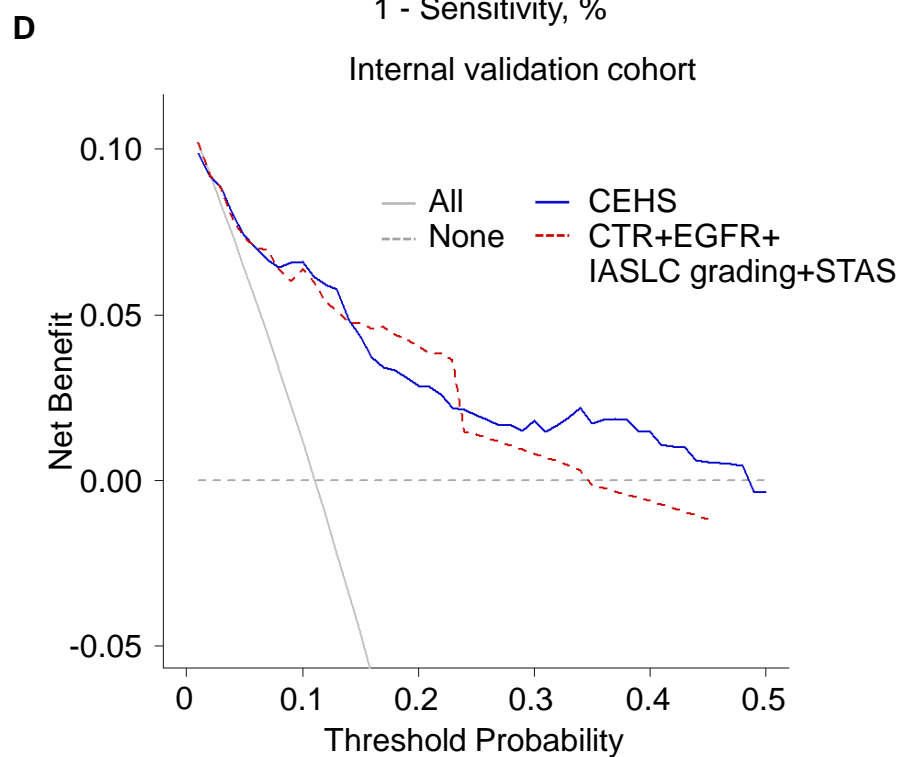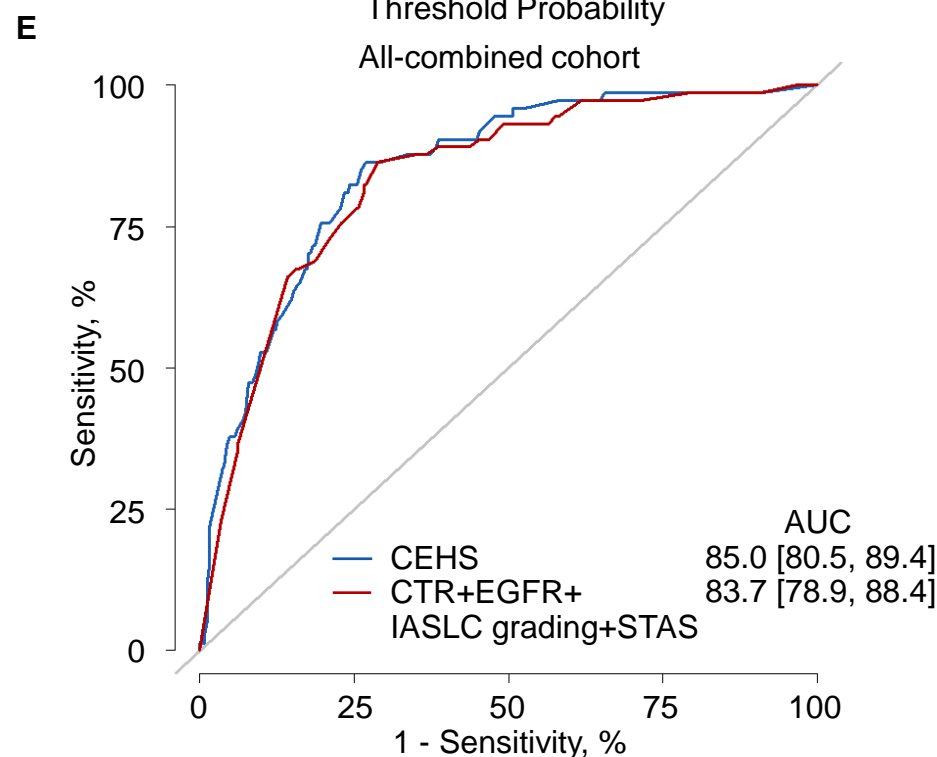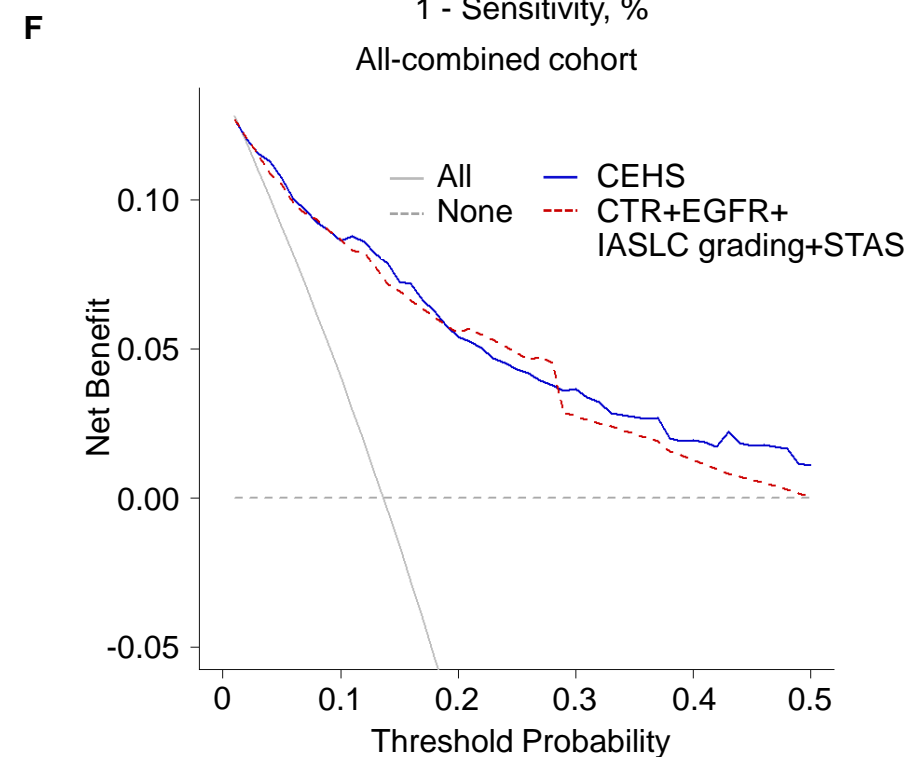

Supplement: Supplementary file 5 — Figure S5: Comparing the two models, the ROC and DAC results showed that the contribution of HGP continuous variables to the model is greater than that of HGP fixed values (IASLC grading). In (A, B) the development cohort (n = 272 patients), (C, D) the internal validation cohort (n = 272 patients) and (E, F) the all‐combined cohort (n = 544 patients), ROC curves and DAC showed the CEHS model was better in predicting the risk of recurrence at 5‐year than the model (including CTR, EGFR, IASLC grading and STAS). (CTR, consolidation tumor ratio; DCA, decision curve analysis; EGFR, epidermal growth factor receptor; HGP, high grade patterns; ROC, receiver operating characteristic; STAS, spread through air spaces). [file TCA-17-e70291-s006.pdf]

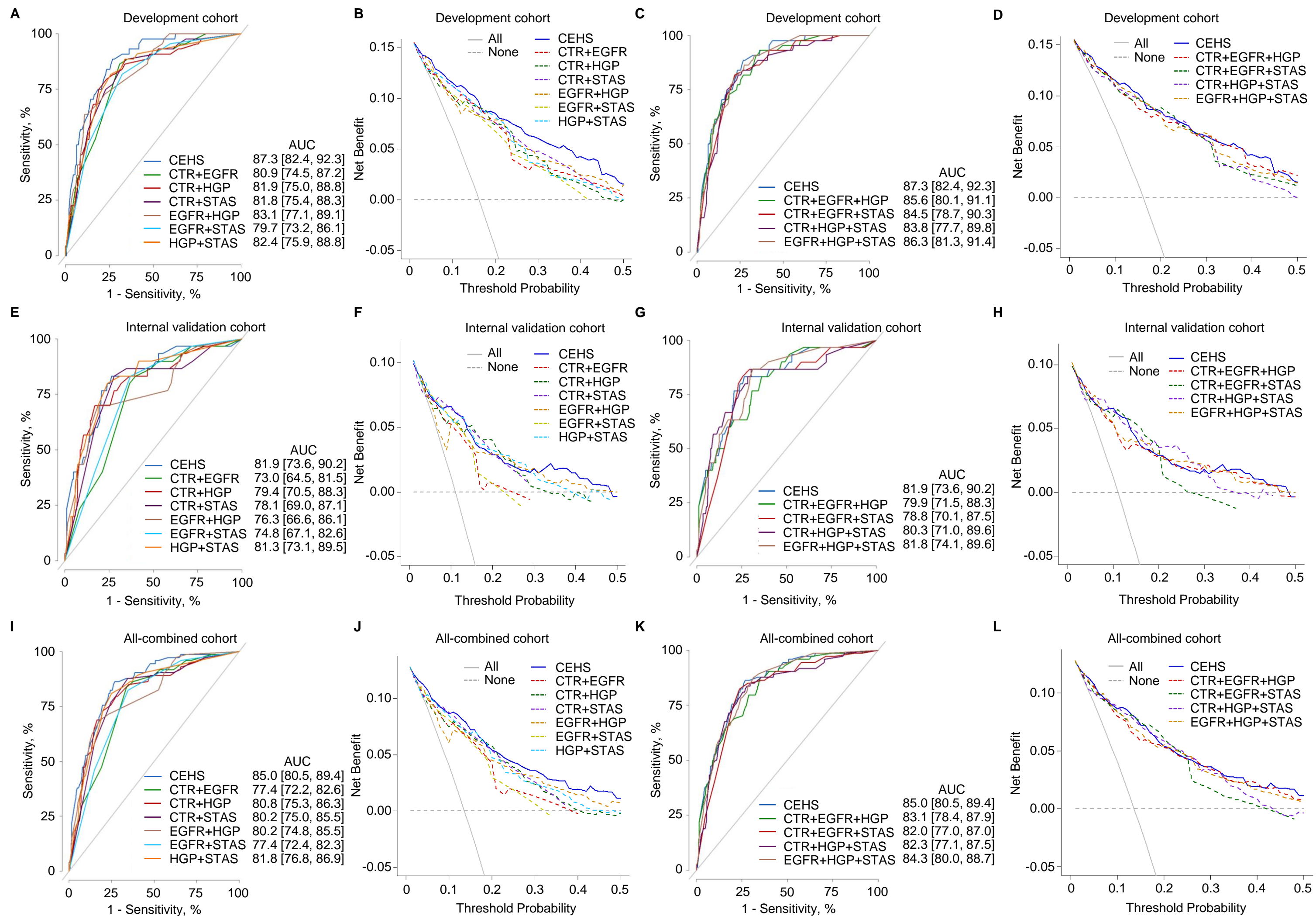

Supplement: Supplementary file 6 — Figure S6: In the development cohort (n = 272 patients), the internal validation cohort (n = 272 patients) and the all‐combined cohort (n = 544 patients), ROC curves and DAC showed the CEHS model was the best model in predicting the risk of recurrence at 5‐year than (A, B, E, F, I, and J) two‐variables Cox models, and (C, D, G, H, K, and L) three‐variables Cox models. (CTR, consolidation tumor ratio; DCA, decision curve analysis; EGFR, epidermal growth factor receptor; HGP, high grade patterns; ROC, receiver operating characteristic; STAS, spread through air spaces). [file TCA-17-e70291-s007.pdf]

# Risk Points

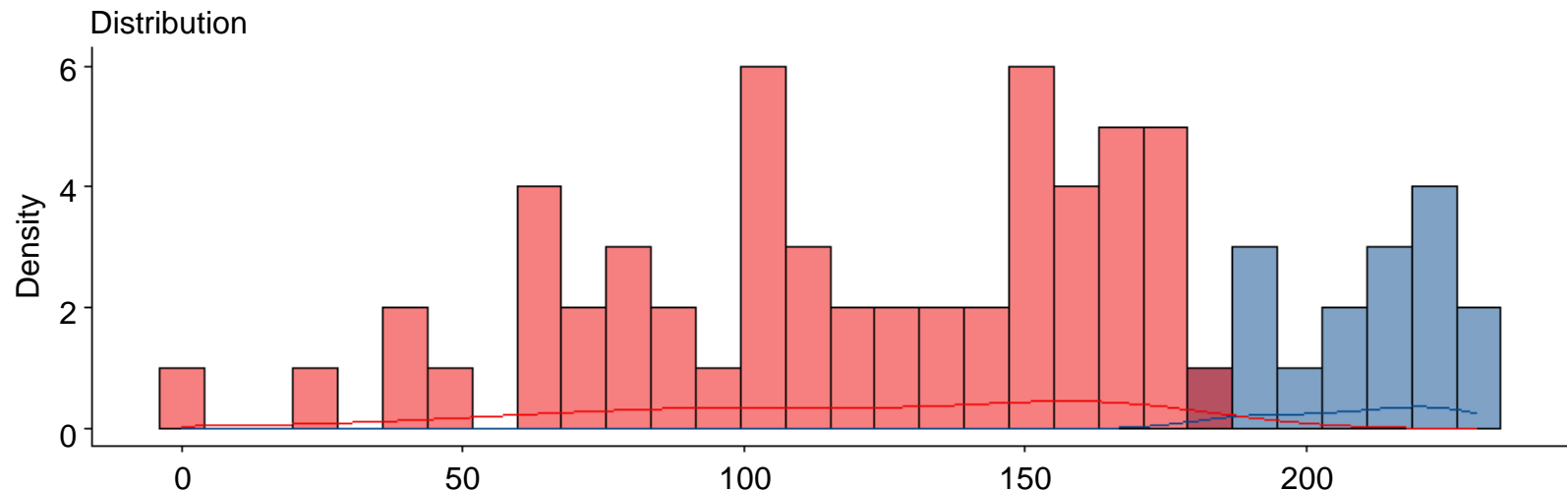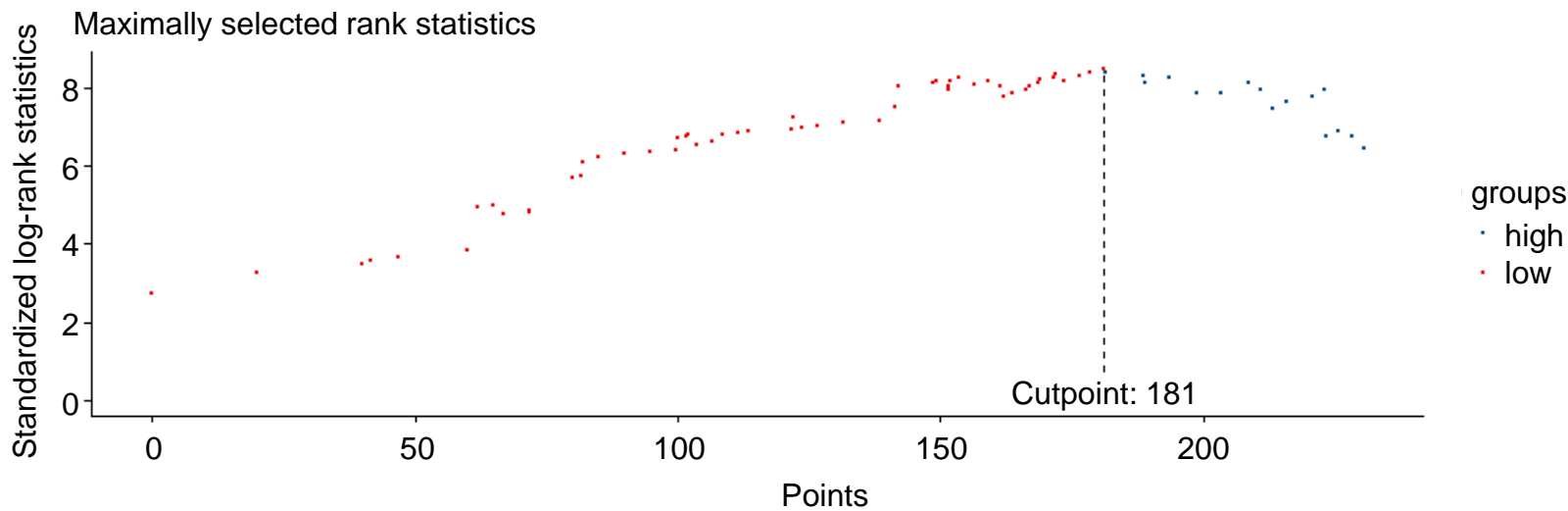

Supplement: Supplementary file 7 — Figure S7: The optimal cutoff value for risk points based on maximum selection logarithmic rank statistics is 181. [file TCA-17-e70291-s004.pdf]
