# Supplementary material for: Radiological, Molecular, and Pathological Factors Unite: A Model for Predicting Recurrence‐Free Survival in Pathological Stage I Lung Adenocarcinoma
Source: Thorac Cancer. 2026 Apr 23;17(8):e70291. doi: 10.1111/1759-7714.70291 (PMC13104728; doi:10.1111/1759-7714.70291)

Dynamic Nomogram

CTR

5

EGFR

Mutation

HGP

1

STAS

Present

☑ Predicted Survival at this Follow Up:

follow.time

60

☑ Alpha blending (transparency)

Predict

Press Quit to exit the application

Quit

Survival plot | Predictive Survival | Numerical Summary | Model Summary

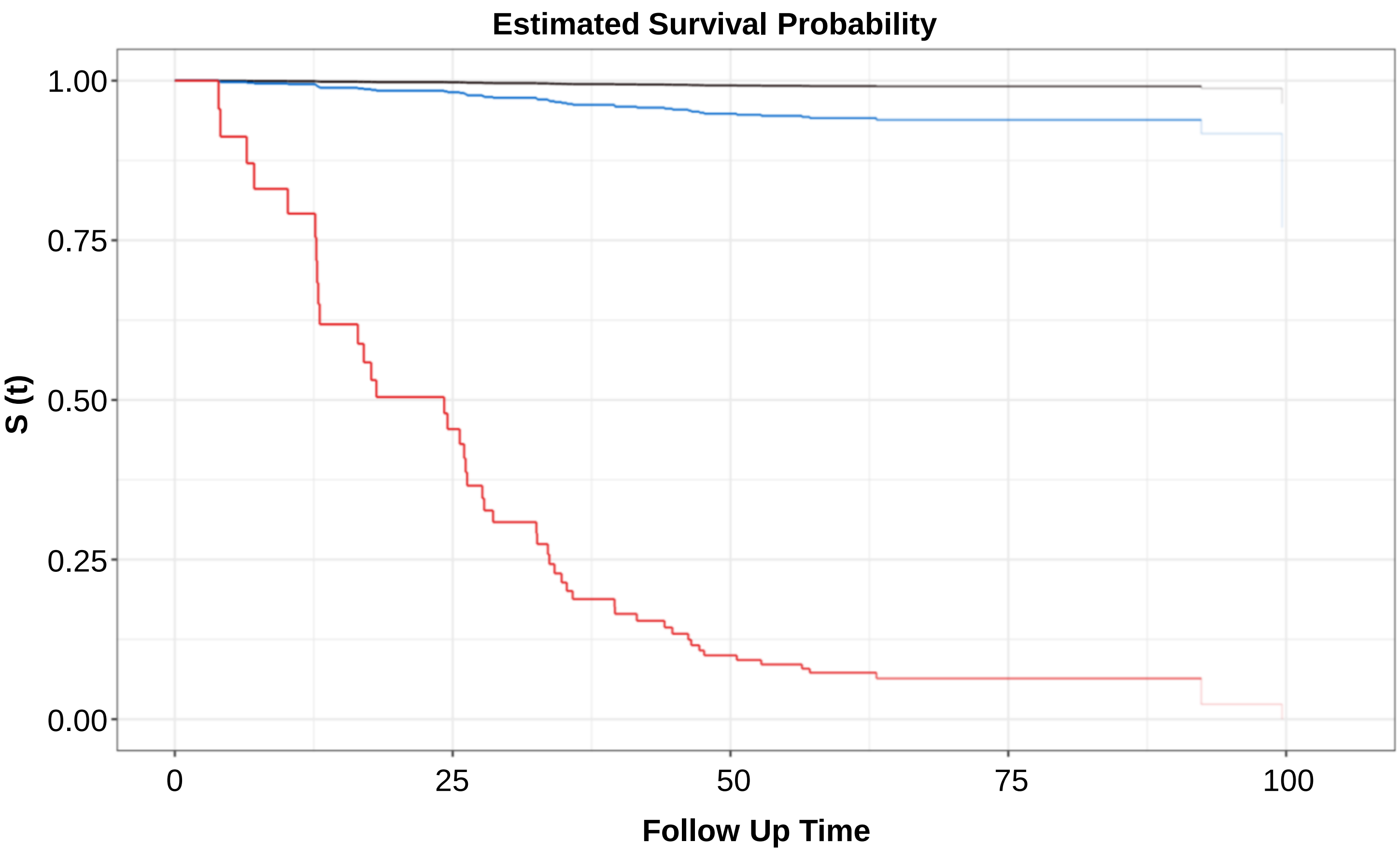

Supplement: Supplementary file 8 — Figure S8: An online calculator is available at: https://zenghui.shinyapps.io/dynnomapp/. (CTR, consolidation tumor ratio; EGFR, epidermal growth factor receptor; HGP, high grade patterns; STAS, spread through air spaces). [file TCA-17-e70291-s005.pdf]
