# Supplementary material for: Radiological, Molecular, and Pathological Factors Unite: A Model for Predicting Recurrence‐Free Survival in Pathological Stage I Lung Adenocarcinoma
Source: Thorac Cancer. 2026 Apr 23;17(8):e70291. doi: 10.1111/1759-7714.70291 (PMC13104728; doi:10.1111/1759-7714.70291)
Supplement: Supplementary file 9 — Table S1: The variables included in this study have been reported in previous literature to have prognostic value for patients with LUAD. Table S2: Multivariable Cox regression models for recurrence risk among patients with p‐stage I LUAD in the development cohort based on a stepdown backward selection with Stepwise Removal of Variables (n = 272). Table S3: Clinical characteristics in the overall, development, and internal validation cohorts according to our newly developed risk groups. [file TCA-17-e70291-s003.docx]

**Supplementary tables**

| **Supplementary Table S1. The variables included in this study have been reported in previous literature to have prognostic value for patients with LUAD** | | | | | | |
| --- | --- | --- | --- | --- | --- | --- |
| **Study** | **Published year** | **Study period** | **Geographic region** | **Stage** | **Outcome** | **Variables related to outcomes** |
| Warth et al. | 2012 | 2002-2008 | Germany | I-IV (7th pTNM stage) | recurrence, death | age, gender |
| Hung et al. | 2013 | 2001-2008 | China | I (7th pTNM stage) | recurrence, death | age, PPPBG system |
| Hung et al. | 2014 | 2004-2010 | China | I-III (7th pTNM stage) | recurrence, death | 7th pT satge, 7th pTNM stage, PPPBG system |
| Ujiie et al. | 2015 | 1999-2009 | America | I (7th pTNM stage) | recurrence, death | gender, scope of operation, 7th pT satge, 7th pTNM stage, PPPBG system |
| Takamochi et al. | 2017 | 2010-2014 | Japan | I-III (7th pTNM stage) | recurrence, death | age, gender, smoking history, EGFR status |
| Nishii et al. | 2017 | 2006-2008 | Japan | I (7th pTNM stage) | recurrence, death | EGFR status |
| Dai et al. | 2017 | 2009-2010 | China | I (8th pTNM stage) | recurrence, death | gender, scope of operation, STAS |
| Zhou et al. | 2018 | 2006-2011 | Japan | I-IV (8th pTNM stage) | progression, death | age, gender, tumor site |
| Ye et al. | 2019 | 2008-2014 | China | IA (8th cTNM stage) | recurrence, death | gender |
| Hattori et al. | 2019 | 2008-2012 | Japan | I-IV (8th cTNM stage) | recurrence, death | CTR |
| Ito et al. | 2020 | 2010-2016 | Japan | I-IIIA (8th pTNM stage) | recurrence | 8th pTNM stage, EGFR status |
| Vaghjiani et al. | 2020 | 2000-2014 | America | IA (8th cTNM stage) | recurrence, death | gender, STAS, PPPBG system |
| Moreira et al. | 2020 | NA | America and Australia | I-III (8th pTNM stage) | recurrence, death | STAS, HGP |
| Kim et al. | 2021 | 2005-2017 | Korea | I-IIIA (8th pTNM stage) | recurrence | age, gender, 8th pTNM stage |
| Xi et al. | 2021 | 2011-2016 | China | IA (8th cTNM stage) | recurrence | CTR |
| Jeon et al. | 2021 | 2010-2015 | Korea | IA (8th pTNM stage) | recurrence | gender, smoking history, PPPBG system |
| Chae et al. | 2021 | 2009-2016 | Korea | IA (8th pTNM stage) | recurrence, death | smoking history, STAS |
| Wang et al. | 2021 | 2011-2015 | China | I-III (8th pTNM stage) | recurrence, death | gender, spiculation, pleural retraction, vacuole |
| Hou et al. | 2022 | 2015 | China | I (8th pTNM stage) | recurrence, death | IASLC grading, 8th pT satge, 8th pTNM stage |
| Fujikawa et al. | 2022 | 2016-2018 | Japan | I-III (8th pTNM stage) | recurrence, death | age, gender, smoking history, 8th pTNM stage, IASLC grading |
| Jeong et al. | 2022 | 2010-2015 | Korea | I-II (8th pTNM stage) | recurrence, death | age, gender, smoking history, 8th pTNM stage, tumor site |
| LUAD = lung adenocarcinoma; CTR = consolidation tumor ratio; EGFR = epidermal growth factor receptor; 8th =eighth edition; pT = pathological Tumor; pTNM = pathological Tumor-Node-Metastasis; PPPBG = previous predominant pattern-based grade; IASLC = International Association for the Study of Lung Cancer; HGP = high grade patterns; STAS = spread through air spaces. | | | | | | |

| **Supplementary Table S2. Multivariable Cox regression models for recurrence risk among patients with p-stage I LUAD in the development cohort based on a stepdown backward selection with Stepwise Removal of Variables (n = 272)** | | | | | | | | | | | | | | | | | | | | | | |  |
| --- | --- | --- | --- | --- | --- | --- | --- | --- | --- | --- | --- | --- | --- | --- | --- | --- | --- | --- | --- | --- | --- | --- | --- |
| **Variable** | **No. of participants** | | |  | **HR (95% CI)** | | | | | | | | | | | | | | | | | |  |
|  | **All (n = 272)** | **Cases (n = 47)** | |  | **model 1 (AIC = 443.40)** | | **model 2 (AIC = 443.40)** | **model 3 (AIC = 440.47)** | | **model 4 (AIC = 438.86)** | | **model 5 (AIC = 436.07)** | | **model 6 (AIC = 435.81)** | | **model 7 (AIC = 437.86)** | | **model 8**  **(AIC = 441.16)** | | **model 9**  **(AIC = 445.89)** | **model 10**  **(AIC = 447.22)** | |  |
| Smoking history |  |  | |  |  | |  |  | |  | |  | |  | |  | |  | |  |  | |  |
| never | 202 | 28 | |  | reference | | reference | reference | | reference | | reference | | NA | | NA | | NA | | NA | NA | |  |
| ever | 70 | 19 | |  | 1.46 (0.79 - 2.70) | | 1.46 (0.79 - 2.70) | 1.53 (0.83 - 2.83) | | 1.53 (0.83 - 2.82) | | 1.52 (0.82 - 2.81) | | NA | | NA | | NA | | NA | NA | |  |
| CTR | 272 | 47 | |  | 1.36 (0.98 - 1.89) | | 1.36 (0.98 - 1.89) | 1.46 (1.06 - 2.01) | | 1.49 (1.09 - 2.04) | | 1.47 (1.09 - 1.99) | | 1.46 (1.08 - 1.98) | | 1.57 (1.17 - 2.10) | | NA | | 1.78 (1.32 - 2.39) | 1.43 (1.06 - 1.93) | |  |
| EGFR |  |  | |  |  | |  |  | |  | |  | |  | |  | |  | |  |  | |  |
| wild | 148 | 18 | |  | reference | | reference | reference | | reference | | reference | | reference | | reference | | reference | | reference | NA | |  |
| mutation | 124 | 29 | |  | 2.89 (1.49 - 5.56) | | 2.88 (1.49 - 5.56) | 3.31 (1.74 - 6.29) | | 3.33 (1.75 - 6.36) | | 3.21 (1.69 - 6.09) | | 3.24 (1.69 - 6.20) | | 3.89 (2.04 - 7.41) | | 3.13 (1.64 - 5.97) | | 2.40 (1.32 - 4.39) | NA | |  |
| PPPBG system |  |  | |  |  | |  |  | |  | |  | |  | |  | |  | |  |  | |  |
| high grade | 32 | 1 | |  | reference | | reference | reference | | reference | | NA | | NA | | NA | | NA | | NA | NA | |  |
| intermediate grade | 220 | 34 | |  | 1.52 (0.14 - 16.66) | | 1.52 (0.14 - 16.67) | 1.77 (0.17 - 18.44) | | 1.25 (0.16 - 9.99) | | NA | | NA | | NA | | NA | | NA | NA | |  |
| low grade | 20 | 12 | |  | 3.26 (0.23 - 46.68) | | 3.26 (0.23 - 46.68) | 2.96 (0.23 - 38.16) | | 2.02 (0.21 - 19.18) | | NA | | NA | | NA | | NA | | NA | NA | |  |
| IASLC grading |  |  | |  |  | |  |  | |  | |  | |  | |  | |  | |  |  | |  |
| grade 1 | 32 | 1 | |  | reference | | reference | reference | | NA | | NA | | NA | | NA | | NA | | NA | NA | |  |
| grade 2 | 163 | 12 | |  | 0.69 (0.22 - 2.18) | | 0.69 (0.22 - 2.18) | 0.69 (0.22 - 2.18) | | NA | | NA | | NA | | NA | | NA | | NA | NA | |  |
| grade 3 | 77 | 34 | |  | NA | | NA | NA | | NA | | NA | | NA | | NA | | NA | | NA | NA | |  |
| 8th pT stage |  |  | |  |  | |  |  | |  | |  | |  | |  | |  | |  |  | |  |
| T1a | 33 | 1 | |  | reference | | reference | NA | | NA | | NA | | NA | | NA | | NA | | NA | NA | |  |
| T1b | 140 | 16 | |  | 1.23 (0.15 - 10.07) | | 1.23 (0.15 - 10.07) | NA | | NA | | NA | | NA | | NA | | NA | | NA | NA | |  |
| T1c | 77 | 19 | |  | 2.09 (0.25 - 17.48) | | 2.09 (0.25 - 17.48) | NA | | NA | | NA | | NA | | NA | | NA | | NA | NA | |  |
| T2a | 22 | 11 | |  | 2.38 (0.25 - 22.27) | | 2.38 (0.25 - 22.27) | NA | | NA | | NA | | NA | | NA | | NA | | NA | NA | |  |
| 8th pTNM stage |  |  | |  |  | |  |  | |  | |  | |  | |  | |  | |  |  | |  |
| IA | 250 | 36 | |  | reference | | NA | NA | | NA | | NA | | NA | | NA | | NA | | NA | NA | |  |
| IB | 22 | 11 | |  | NA | | NA | NA | | NA | | NA | | NA | | NA | | NA | | NA | NA | |  |
| HGP | 272 | 47 | |  | 2.18 (0.40 - 11.79) | | 2.18 (0.40 - 11.79) | 2.80 (0.57 - 13.72) | | 3.75 (1.02 - 13.76) | | 5.42 (1.84 - 15.98) | | 6.51 (2.28 - 18.57) | | 9.14 (3.43 - 24.34) | | 12.81 (4.94 - 33.18) | | NA | 3.69 (1.45 - 9.40) | |  |
| STAS |  |  | |  |  | |  |  | |  | |  | |  | |  | |  | |  |  | |  |
| absence | 165 | 10 | |  | reference | | reference | reference | | reference | | reference | | reference | | NA | | reference | | reference | reference | |  |
| present | 107 | 37 | |  | 2.05 (0.83 - 5.03) | | 2.05 (0.83 - 5.03) | 1.98 (0.80 - 4.89) | | 2.23 (0.99 - 5.02) | | 2.24 (1.01 - 4.99) | | 2.21 (0.99 - 4.95) | | NA | | 3.10 (1.40 - 6.89) | | 3.36 (1.58 - 7.12) | 2.96 (1.36 - 6.41) | |  |
| p-stage = pathological stage; LUAD = lung adenocarcinoma; No. = number; HR = hazard ratio; CI = confidence interval; AIC = Akaike Information Criterion; CTR = consolidation tumor ratio; EGFR = epidermal growth factor receptor; 8th = eighth edition; pT = pathological Tumor; pTNM = pathological Tumor-Node-Metastasis; PPPBG = previous predominant pattern-based grade; IASLC = International Association for the Study of Lung Cancer; HGP = high grade patterns; STAS = spread through air spaces. Model 6 was the optimal model (the lowest AIC = 435.8). | | | | | | | | | | | | | | | | | | | | | | |  |
| **Supplementary Table S3. Clinical characteristics in the overall, development, and internal validation cohorts according to our newly developed risk groups** | | | | | | | | | | | | | | | | | | | | | | | |
| **Variable** | | | **Development cohort (n = 272)** | | | | | | | | **Internal Validation cohort (n = 272)** | | | | | | **All-combined cohort (n = 544)** | | | | | | |
|  |  |  | **low (n = 209)** | | | **high (n = 63)** | | | ***P* - value** | | **low (n = 206)** | | **high (n = 66)** | | ***P* - value** | | **low (n = 415)** | | **high (n = 129)** | | | ***P* - value** | |
| CTR ᵃ | | |  | | |  | | | < 0.001 | |  | |  | | < 0.001 | |  | |  | | | < 0.001 | |
| 0 | | | 65 (31.1) | | | 0 (0.0) | | |  | | 55 (26.7) | | 0 (0.0) | |  | | 120 (28.9) | | 0 (0.0) | | |  | |
| < 25% | | | 25 (12.0) | | | 0 (0.0) | | |  | | 39 (18.9) | | 0 (0.0) | |  | | 64 (15.4) | | 0 (0.0) | | |  | |
| 25 - 50% | | | 14 (6.7) | | | 0 (0.0) | | |  | | 17 (8.3) | | 1 (1.5) | |  | | 31 (7.5) | | 1 (0.8) | | |  | |
| 50 - 75% | | | 26 (12.4) | | | 0 (0.0) | | |  | | 29 (14.1) | | 0 (0.0) | |  | | 55 (13.3) | | 0 (0.0) | | |  | |
| ≥ 75% | | | 49 (23.4) | | | 18 (28.6) | | |  | | 40 (19.4) | | 18 (27.3) | |  | | 89 (21.4) | | 36 (27.9) | | |  | |
| 1 | | | 30 (14.4) | | | 45 (71.4) | | |  | | 26 (12.6) | | 47 (71.2) | |  | | 56 (13.5) | | 92 (71.3) | | |  | |
| EGFR ᵃ | | |  | | |  | | | 0.002 | |  | |  | | 0.150 | |  | |  | | | 0.001 | |
| wild | | | 125 (59.8) | | | 23 (36.5) | | |  | | 101 (49.0) | | 25 (37.9) | |  | | 226 (54.5) | | 48 (37.2) | | |  | |
| mutation | | | 84 (40.2) | | | 40 (63.5) | | |  | | 105 (51.0) | | 41 (62.1) | |  | | 189 (45.5) | | 81 (62.8) | | |  | |
| HGP ᵇ | | | 0.0 (0.0, 0.1) | | | 0.5 (0.2, 0.9) | | | < 0.001 | | 0.0 (0.0, 0.1) | | 0.4 (0.2, 0.9) | | < 0.001 | | 0.0 (0.0, 0.1) | | 0.5 (0.2, 0.9) | | | < 0.001 | |
| STAS ᵃ | | |  | | |  | | | < 0.001 | |  | |  | | < 0.001 | |  | |  | | | < 0.001 | |
| absence | | | 159 (76.1) | | | 6 (9.5) | | |  | | 160 (77.7) | | 2 (3.0) | |  | | 319 (76.9) | | 8 (6.2) | | |  | |
| present | | | 50 (23.9) | | | 57 (90.5) | | |  | | 46 (22.3) | | 64 (97.0) | |  | | 96 (23.1) | | 121 (93.8) | | |  | |
| 8th pTNM stage ᶜ | | |  | | |  | | | < 0.001 | |  | |  | | < 0.001 | |  | |  | | | < 0.001 | |
| IA | | | 200 (95.7) | | | 50 (79.4) | | |  | | 200 (97.1) | | 54 (81.8) | |  | | 400 (96.4) | | 104 (80.6) | | |  | |
| IB | | | 9 (4.3) | | | 13 (20.6) | | |  | | 6 (2.9) | | 12 (18.2) | |  | | 15 (3.6) | | 25 (19.4) | | |  | |
| PPPBG system ᶜ | | |  | | |  | | | < 0.001 | |  | |  | | < 0.001 | |  | |  | | | < 0.001 | |
| low grade | | | 32 (15.3) | | | 0 (0.0) | | |  | | 36 (17.5) | | 0 (0.0) | |  | | 68 (16.4) | | 0 (0.0) | | |  | |
| Intermediate grade | | | 177 (84.7) | | | 43 (68.3) | | |  | | 155 (75.2) | | 15 (22.7) | |  | | 309 (74.5) | | 24 (18.6) | | |  | |
| high grade | | | 0 (0.0) | | | 20 (31.7) | | |  | | 15 (7.3) | | 51 (77.3) | |  | | 38 (9.2) | | 105 (81.4) | | |  | |
| IASLC grading ᶜ | | |  | | |  | | | < 0.001 | |  | |  | | < 0.001 | |  | |  | | | < 0.001 | |
| grade 1 | | | 32 (15.3) | | | 0 (0.0) | | |  | | 36 (17.5) | | 0 (0.0) | |  | | 68 (16.4) | | 0 (0.0) | | |  | |
| grade 2 | | | 154 (73.7) | | | 9 (14.3) | | |  | | 168 (81.6) | | 41 (62.1) | |  | | 345 (83.1) | | 84 (65.1) | | |  | |
| grade 3 | | | 23 (11.0) | | | 54 (85.7) | | |  | | 2 (1.0) | | 25 (37.9) | |  | | 2 (0.5) | | 45 (34.9) | | |  | |
| Recurrence ᵃ | | |  | | |  | | | < 0.001 | |  | |  | | < 0.001 | |  | |  | | | < 0.001 | |
| no | | | 195 (93.3) | | | 30 (47.6) | | |  | | 194 (94.2) | | 46 (69.7) | |  | | 389 (93.7) | | 76 (58.9) | | |  | |
| yes | | | 14 (6.7) | | | 33 (52.4) | | |  | | 12 (5.8) | | 20 (30.3) | |  | | 26 (6.3) | | 53 (41.1) | | |  | |
| Follow time ᵇ ᶜ | | | 81.6 (61.6, 89.2) | | | 56.4 (30.6, 67.2) | | | < 0.001 | | 75.3 (60.8, 88.9) | | 61.6 (44.2, 82.3) | | < 0.001 | | 76.8 (61.2, 89.1) | | 60.5 (33.7, 78.9) | | | < 0.001 | |
| ᵃ number (%); ᵇ median (IQR); ᶜ (month); IQR = interquartile range; CTR = consolidation tumor ratio; EGFR = epidermal growth factor receptor; HGP = high grade patterns; STAS = spread through air spaces; 8th = eighth edition; pTNM = pathological Tumor-Node-Metastasis; PPPBG = previous predominant pattern-based grade; IASLC = International Association for the Study of Lung Cancer. | | | | | | | | | | | | | | | | | | | | | | | |
